# On the reliability of behavioral measures of cognitive control: retest reliability of task-inhibition effect, task-preparation effect, Stroop-like interference, and conflict adaptation effect

**DOI:** 10.1007/s00426-021-01627-x

**Published:** 2021-12-18

**Authors:** Stefanie Schuch, Andrea M. Philipp, Luisa Maulitz, Iring Koch

**Affiliations:** Institute of Psychology, RWTH Aachen University, Jaegerstrasse 17/19, 52066 Aachen, Germany

## Abstract

This study examined the reliability (retest and split-half) of four common behavioral measures of cognitive control. In Experiment 1 (*N* = 96), we examined *N *– 2 task repetition costs as a marker of task-level inhibition, and the cue-stimulus interval (CSI) effect as a marker of time-based task preparation. In Experiment 2 (*N* = 48), we examined a Stroop-like face-name interference effect as a measure of distractor interference control, and the sequential congruency effect (“conflict adaptation effect”) as a measure of conflict-triggered adaptation of cognitive control. In both experiments, the measures were assessed in two sessions on the same day, separated by a 10 min-long unrelated filler task. We observed substantial experimental effects with medium to large effect sizes. At the same time, split-half reliabilities were moderate, and retest reliabilities were poor, for most measures, except for the CSI effect. Retest reliability of the Stroop-like effect was improved when considering only trials preceded by congruent trials. Together, the data suggest that these cognitive control measures are well suited for assessing group-level effects of cognitive control. Yet, except for the CSI effect, these measures do not seem suitable for reliably assessing interindividual differences in the strength of cognitive control, and therefore are not suited for correlational approaches. We discuss possible reasons for the discrepancy between robustness at the group level and reliability at the level of interindividual differences.

**Supplementary Information:**

The online version contains supplementary material available at 10.1007/s00426-021-01627-x.

## Introduction

Human behavior is characterized by its high adaptability and flexibility, so that goals can be achieved even if environmental factors create interference, or goals can be internally shifted despite unchanged environmental factors. The cognitive processes underlying this flexibility have recently been examined with respect to the notion of “cognitive control” (sometimes also called “executive functions”), which generally denotes the ability of humans to intentionally shift goals, update working-memory content, monitor own responses, and inhibit processing of distractors, unwanted thoughts, or prepotent but inappropriate responses.

Specifically, two theoretical frameworks have gained much interest in research of cognitive control over the last two decades. First, based on investigations of shared variance among sets of intercorrelated tasks, Miyake et al. (2000) proposed a framework that postulates three general aspects of cognitive control: shifting of task-set, monitoring and updating of working-memory, and inhibition of prepotent response tendencies (see Karr et al., 2018, for a recent review). Second, based on work on sequential modulation of well-established interference effects (i.e., the “sequential congruency effect”; Egner, 2007, for a review), Botvinick et al., (2001; Botvinick et al., 2004) developed a conflict-monitoring account, proposing that response conflict is internally monitored, and the detection of conflict triggering an upregulation of selective attention. Both frameworks are based on empirical “signature” effects, such as task-switch costs for the shifting component of cognitive control (for reviews, see, e.g., Kiesel et al., 2010; Koch et al., 2018; Monsell, 2003; Vandierendonck et al., 2010), the well-known Stroop effect as an indicator of the degree of attentional selectivity (e.g., Stroop, 1935; see MacLeod, 1991), and its sequential modulation as an indicator of conflict-triggered control adjustments (see also Schuch et al., 2019, for a recent discussion of sequential interference effects in multitasking paradigms).

Theoretical frameworks have a strong heuristic influence and allow researchers to derive empirical hypotheses to be tested in experimental studies. In fact, the two mentioned frameworks have inspired many empirical studies and theoretical developments. In the present study, we focus on a particular research question that is derived primarily from recent attempts to understand cognitive control as a construct that can be decomposed, and assuming that the components can be measured separately. The availability of valid measures of subcomponents of cognitive control would be of tremendous importance for many applied areas, such as psychological assessment and clinical psychology. In these domains, the assessment of interindividual differences in subcomponents of cognitive control is of vital importance. Valid and reliable measures of cognitive control components are also a necessary precondition for addressing the long-standing question of whether cognitive control functions are domain-general (i.e., whether an up- or down-regulation of control in one task also affects the level of control in another task) or domain-specific (i.e., the level of control is regulated separately for different tasks, or domains; see Braem et al., 2014; Egner, 2008, for reviews on the domain-specificity versus domain-generality of the sequential congruency effect).

Yet, recently, doubts about the reliability of interindividual differences in many cognitive control measures have emerged (e.g., Miyake & Friedman, 2012; Paap & Sawi, 2016; Rey-Mermet et al., 2018). As it turns out, cognitive control measures that prove stable and reliable when measured at a group level (i.e., effects that have been replicated in many experimental studies using different participant samples, e.g. the “Many-Labs Project”, Klein et al., 2014, and follow-up projects), do not necessarily show sufficient split-half and retest reliability when taken to assess interindividual differences (as is the case with correlational approaches, such as structural equation modeling). This puzzling discrepancy between reliability on a group level (i.e., the probability of replicating a group-level effect in a new sample of participants) and reliability on the level of interindividual differences (i.e., split-half and retest reliability) has recently been termed the “reliability paradox” (Hedge et al., 2018a, 2018b, 2020).

In the present study, we aimed to examine the split-half and retest reliability of some prominent cognitive control measures. In Experiment 1, we examined the reliability of two important effects in task switching: *N *− 2 task repetition costs and general cue-based task preparation benefit. In Experiment 2, we examined the reliability of two widely used effects in single-task paradigms: a Stroop-like effect (using a face-name interference paradigm) and its sequential modulation (sequential congruency effect, or “conflict adaptation effect”). All of these effects have previously been examined in several studies in our own labs, and have proven to be robust effects when measured at the group level (for reviews of the task-switching related effects, see Gade et al., 2014; Koch et al., 2010; Koch et al., 2018; for congruency and sequential congruency effects in single-task paradigms, see e.g., Schuch & Koch, 2015, Schuch et al., 2017; for review, see Schuch et al., 2019). In the following, we will briefly discuss the theoretical background of a) the two task-switching measures and b) the two single-task measures of cognitive control.

### Cognitive control measures in task switching

In the structural-equation modeling approach presented by Miyake et al. (2000), three subcomponents of cognitive control were identified as latent variables: “shifting of task-sets”, “monitoring and updating of working memory”, and “inhibition of prepotent response tendencies”. Later empirical work using structural equation modeling confirmed the task-shifting and working-memory factors, but not the “inhibition of prepotent responses” factor (see Friedman & Miyake, 2017; Karr et al., 2018; Miyake & Friedman, 2012, for reviews). Instead, Friedman et al. (2008) proposed a “common executive function” factor that partially overlaps with the task-shifting and working-memory factors. They describe this common factor as “the ability to maintain and manage goals, and use those goals to bias ongoing processing” (Friedman & Miyake, 2017, citation from section “5.1.1. Hypothesized functions for the Common EF factor”). The working-memory factor is characterized by the ability to update some of the current working-memory content, while at the same time maintaining other working-memory content for later retrieval. This factor is measured by memory tasks that require participants to attend to sequentially presented items from different categories, and later recall the last item from each category.

The task-shifting factor is described as the ability to rapidly replace task-sets in Friedman and Miyake’s (2017) framework, and the authors suggest that participants might differ in the speed of task-set replacement (see also Miyake & Friedman, 2012). The task-shifting factor is measured by cued task-switching paradigms, where the currently relevant task-set changes from trial to trial and is indicated by a task cue that is presented prior to the target stimulus (Meiran, 1996).

One popular measure that can be extracted from task-switching paradigms is the “task-switch cost”, defined as the performance difference between task-switch trials and task-repetition trials in a cued task-switching paradigm. For instance, Friedman and Miyake (2004) tested more than 200 participants with three cued task-switching paradigms, and found good reliability of task-switch costs (with Spearman-Brown corrected split-half reliabilities ranging from *r* = 0.43 to *r* = 0.82). The reliability of task-switch costs has been confirmed in several other studies (e.g., Friedman et al., 2008; Miyake et al., 2000; Paap et al., 2017; Pettigrew & Martin, 2016).

However, it is widely acknowledged that task-switch costs represent a mixture of different effects (see Kiesel et al., 2010; Koch et al., 2018, for reviews). One subcomponent of task-switch costs is task-level inhibition (e.g., Allport & Wylie, 1999; Goschke, 2000). Task-level inhibition can be measured with “*N* − 2 task repetition costs” (e.g., Mayr & Keele, 2000; see Koch et al., 2010, for a review), which is a sequential measure where different kinds of task-switching sequences are compared. For example, Gade and Koch (2005) used three tasks, and in each trial, the task was indicated by an explicit instruction cue. As stimuli, they used colored (red vs. blue) symbols (a digit or a letter) that varied in size (small vs. large), so that there were three varying perceptual dimensions, and the task cue indicated the relevant stimulus dimension for selecting the target attribute (e.g., small vs. large for the size dimension). When the authors analyzed the sequential transitions, they found that sequences of the ABA type (*N* − 2 task repetitions, e.g., color–size–color) resulted in worse performance (e.g., higher reaction time [RT]) than sequences of the CBA type (*N* − 2 switches, e.g. symbol-size-color). The finding of higher RT in the last trial of an ABA versus CBA task sequence speaks in favor of a process that inhibits aspects of the preceding task set when shifting to a new task set; this is because accounts in terms of persisting activation of previously established task representations (task sets) would predict *better* performance for ABA relative to CBA (Mayr & Keele, 2000; see Koch et al., 2018, for a recent discussion). Even though some other effects in task switching have been related to inhibitory processing, *N* − 2 repetition costs arguably represent the most unambiguous case for inhibition in task switching to date (Koch et al., 2010; and see also Grange et al., 2017, for a recent discussion). Yet, even though the experimental evidence for the existence (and replicability on the group level) of *N* − 2 repetition costs in task switching is very robust (i.e., they have been replicated many times with different paradigms and in different participant samples, see Koch et al., 2018, for a recent review), only few studies examined its split-half and retest reliability.

To our knowledge, three studies so far have assessed split-half reliability of *N* − 2 repetition costs. Kowalczyk and Grange (2017) used three different versions of task switching and found split-half reliabilities of *N* − 2 repetition costs between *r* = 0.37 and *r* = 0.60 (these are corrected reliability scores; note that split-half reliability is usually corrected for attenuation by applying the Spearman–Brown correction). Pettigrew and Martin (2016) reported a split-half reliability of *N* − 2 repetition costs of *r* = 0.44, and Rey-Mermet et al. (2018) of *r* = 0.27. One study assessed test–retest reliability of *N* − 2 repetition costs in both a task-switching and a language-switching paradigm, with about one week between test and retest (Timmer et al., 2018). These authors observed a retest reliability of *N* − 2 repetition costs of *r* ≈ 0.40 (both in the task-switching and the language-switching paradigm). Taken together, the available data on the reliability of *N* − 2 repetition costs is scarce and ranging from poor to moderate reliabilities.

Apart from task-switch costs and *N* − 2 repetition costs, another important cognitive-control measure that can be assessed in cued task-switching paradigms is the time-based task-preparation effect. Here we define this effect as the performance difference between trials with short versus long time intervals between task cue and task-specific stimulus (cue-stimulus interval, CSI). For instance, Lawo et al. (2012) observed substantial task-preparation effects that differed between younger and older adults, suggesting that task-preparation ability deteriorates with older age (on a group level). Other aging and developmental studies confirm that the efficiency of task preparation is an important aspect when assessing age-related differences in cognitive control (e.g., Cepeda et al., 2001; Crone et al., 2006; Schuch, 2016; Schuch & Konrad, 2017; Wild-Wall et al., 2007; for reviews, see Gajewski et al., 2018; Kray & Doerrenbaecher, in press; Kray & Ferdinand, 2014). Assuming that the relevant task set becomes activated during the CSI, the performance difference between short and long CSI conditions can be interpreted as reflecting the degree of cue-based activation of the relevant task-set, especially in *N *− 2 repetition cost paradigms where usually every trial is a task switch (e.g., Lawo et al., 2012; Schuch & Grange, 2019; Schuch & Koch, 2003). It is often assumed that task preparation involves activation of the relevant attentional settings and task rules in working memory and builds up gradually over time, such that a longer CSI leads to better task preparation (for reviews, see Kiesel et al., 2010; Koch et al., 2018).

Beyond the general task-preparation effect discussed here (i.e., the reduction of mean RT in trials with long vs. short CSI), considerable research has been carried out focusing on the specific task-preparation effect, denoting the reduction of task-switch costs with long vs. short CSI (see Kiesel et al., 2010; Koch et al., 2018, for reviews). The latter measure is often interpreted as a marker of “advance reconfiguration of task set” (Meiran, 1996; Monsell, 2003; Vandierendonck et al., 2010). Whether such a specific task-preparation effect also occurs with *N *− 2 task repetition costs to date is an unresolved issue. While earlier studies did not find a reduction of *N *− 2 task repetition costs with longer as compared to shorter CSI (e.g., Mayr & Keele, 2000; Schuch & Koch, 2003; see Koch et al., 2010, for review), more recent studies do sometimes report reduced N-2 repetition costs with longer task-preparation time (e.g., Gade & Koch, 2014; Scheil & Kleinsorge, 2014; Schuch & Grange, 2019). The design of the present Experiment 1 allowed us to contribute to this literature, by examining *N *− 2 repetition costs with short vs long CSI on a group level.

While CSI effects are well established on a group level, less attention has been paid to their reliability on the level of interindividual differences. Yet, the general task-preparation effect (i.e., performance improvement with long as compared to short CSI)—if it proves to be reliable—might be a good candidate for investigations of task switching processes from an interindividual-differences perspective. For instance, in the aging literature, age-related differences in task-preparation processes are widely discussed (e.g., Kray & Ferdinand, 2014, for review), but these studies typically compare task-preparation effects on a group level (i.e., comparing a group of younger adults with a group of older adults), such that reliability is usually not in the focus. Yet, the time-based task preparation effect may be suitable for correlational approaches, just as other behavioral indices of task preparation have been used in individual-differences studies (e.g., Wager et al., 2006). For instance, task-preparation effects related to the informativeness of the task cues have been correlated with electrophysiological and neuroimaging markers of task preparation (e.g., Brass & von Cramon, 2004; Karayanidis et al., 2009; see, e.g., Hsieh, 2012; Karayanidis et al., 2010, for reviews). Hence, assessing reliability of task-preparation measures in general, and of the time-based task-preparation effect in particular, might be useful for future investigations of cognitive control from an individual-differences perspective.

### Cognitive control measures in single-task paradigms

Regarding cognitive control measures in single-task context, perhaps the most popular effect is the color-word Stroop effect (i.e., saying the ink color of written color words that are either congruent or incongruent with the ink color they are presented in; see MacLeod, 1991; MacLeod & MacDonald, 2000, for reviews). The Stroop effect is a classic textbook example and popular classroom demonstration of a “conflict task”, where task-relevant and task-irrelevant features interfere, creating some kind of cognitive conflict (e.g., conflict between stimulus features, or conflict between competing responses). It is sometimes explained in terms of an inhibitory process, such as inhibition of distractor processing, or inhibition of inappropriate response tendency (e.g., Friedman & Miyake, 2017; Gärtner & Strobel, 2021; Miyake et al., 2000; Pettigrew & Martin, 2016). Others have argued that the Stroop effect and other conflict tasks do not necessarily reflect inhibitory control (e.g., Paap et al., 2020). Here, we will use the more descriptive terms “distractor interference control” or “control of cognitive conflict”. The Stroop effect has been reported to be quite reliable (with Spearman-Brown corrected split-half reliability often between *r* = 0.80 and *r* = 0.90, see, e.g., Friedman & Miyake, 2004; Rey-Mermet et al., 2018).

Interestingly, despite the high robustness of this experimental effect, when examining the sequential modulation of the Stroop effect (sequential congruency effect, e.g., Egner, 2007), which is typically used to examine conflict adaptation, the split-half reliability of this sequential measure has been found to be very poor, ranging between *r* =  − 0.12 and *r* = 0.08 across three experiments reported by Whitehead et al. (2019). This drop in reliability is at least partly due to the fact that the sequential congruency effect is computed as the difference of a difference score, and therefore has lower reliability than the congruency effect, which is computed as a simple difference score (see Kopp, 2011; Miller & Ulrich, 2013; Whitehead et al., 2019, for considerations on the reliability of difference scores).

While a considerable number of studies assessed split-half reliability of Stroop-like interference effects and task-switching effects, only few studies investigated the retest reliability of such effects. In one recent study, Hedge et al., (2018b) assessed retest reliability of a number of interference effects, including the Stroop effect, with a temporal separation of three weeks between test and retest. They found a retest reliability of *r* = 0.60 and *r* = 0.66 for the Stroop effects in two studies (they did not report retest reliability of the sequential congruency effect). In another study, Paap and Sawi (2016) examined retest reliability of effects in four different tasks, including task switching, over a period of one week and found only moderate reliabilities. For example, for color-shape switching, they found a retest reliability of *r* = 0.62. For the Simon task (which is often considered a conflict task, similar to the Stroop task), they found a retest reliability of only *r* = 0.43.

### The present study

To summarize, several measures of cognitive control that are highly robust when analyzed on the group level in standard experimental paradigms have surprisingly low reliability when taken as a measure of interindividual differences in correlational approaches, for instance, in structural equation modeling. Therefore, more studies are needed that assess the split-half and retest reliability of standard cognitive control measures, to elucidate which of these measures are suitable for individual-differences approaches, and which are not.

In the present study, we assessed the reliability of four standard cognitive control measures. In Experiment 1, we focused on *N *− 2 repetition costs, which are a measure of task-level inhibition (see Koch et al., 2010, for review), and the time-based task-preparation effect (i.e., CSI effect, denoting the finding of improved performance with long as compared to short CSI), which may be considered as a marker of cue-based task-set activation (especially in paradigms with task switches only; e.g., Lawo et al., 2012; Schuch & Grange, 2019; Schuch & Koch, 2003). The design of Experiment 1 also allowed us to explore the potential preparatory modification of *N *− 2 repetition costs by task-preparation time (on a group level).

In Experiment 2, we examined a variant of the Stroop effect. The family of Stroop-like effects is a marker for distractor interference processing, and is sometimes taken as a marker for inhibitory processing; moreover, the sequential modulation of Stroop-like effects has been taken as a hallmark of conflict-triggered adjustments of cognitive control (Botvinick et al., 2001; see also Egner, 2007, 2017; Paap et al., 2019; Schuch et al., 2019, for more recent reviews). Here we used a face-name interference paradigm that resembles paradigms often used in the neuroimaging literature (e.g., Egner & Hirsch, 2005; Gazzaley et al., 2005; O’ Craven, et al., 1999), and has been used in our own lab before (Schuch & Koch, 2015; Schuch et al., 2017).

For these four measures of cognitive control, we report the group-level effects (i.e., the average effects across all participants), as well as their split-half and retest reliability. In both experiments, the respective effects were measured using standard experimental paradigms in a first and second session on the same day, which were separated by a short unrelated filler task. Then, participants performed the same experiment again in a second session (i.e., on the same day). We first report the group-level effects as obtained with a standard analysis of variance (ANOVA), with first vs. second session as an independent within-subjects variable. Then, we report split-half reliability (correlation between odd and even trials) and retest reliability (correlation between first and second session) for each of the effects.

### Methodological considerations: number of participants and number of trials per condition

To get reliable estimates for correlations, two issues are important: first, there needs to be a large enough number of participants—for instance, to reliably detect medium-sized correlations, a sample of *N* = 85 or larger is necessary (Cohen, 1992). With smaller sample sizes, correlation estimates are very variable (Schönbrodt & Perugini, 2013).

Second, and perhaps even more importantly, the number of experimental trials that provide the basis for computation of the experimental effects play a crucial role (Green et al., 2016; Rouder & Haaf, 2019). With small trial numbers, the estimates of the experimental effects are variable, which leads to attenuated correlations between the experimental effects from different conditions. One remedy to this issue is to apply the Spearman-Brown correction formula (Spearman, 1904), which corrects for a reduction of test length (i.e., of trial numbers in the case of experimental effects).^1^ The estimates of split-half reliabilities of experimental effects are often Spearman–Brown corrected, to compensate for halving the “test length” by splitting trials into odd versus even trials. Note, however, that “test length” may vary considerably across experimental paradigms. When assessing, e.g., the Stroop effect, some researchers might use a paradigm with as little as 20 trials per condition, while others might use a different paradigm with, say, 100 trials per condition. Usually, researchers do not pay much attention to the number of trials that provide the basis for computing the experimental effect. Rouder and Haaf (2019) therefore suggested to calculate reliabilities of experimental effects for the case of infinitely large trial numbers. They did so by applying linear mixed models, and including trial-by-trial variability as an additional random factor in the model. They re-analyzed the data from Hedge et al., (2018b), and found retest reliabilities of around *r* = 0.70 for both Stroop and Flanker effect (as opposed to retest reliabilites of *r* = 0.55 and *r* = 0.50 when correlating the effects from first and second session without accounting for trial-by-trial variability). In a similar vein, Whitehead et al. (2020) re-analyzed data from Whitehead et al. (2019), and observed slightly larger split-half reliabilities for Stroop, Flanker, and Simon effects when using linear mixed models that account for trial-by-trial variability (split-half reliabilities ranging between *r* = 0.57 and *r* = 0.65) than when correlating the effects between odd and even trials without accounting for trial-by-trial variability (split-half reliabilities ranging between *r* = 0.31 and *r* = 0.61). Hence, it is important to always consider the number of trials per condition (or to extrapolate to the large-trial limit) when estimating split-half and test–retest reliabilities of experimental effects. Here, we considered the number of trials per condition when comparing reliability scores of different kinds (retest, split-half), and when comparing reliability measures across different studies.

The large-trial limit might be regarded as the “ideal case” for computing reliabilities; however, there are assets and drawbacks for designing experiments with large trial numbers. A potential disadvantage is that the longer the experiment, the more pronounced the influence of practice effects, and the more likely the cognitive tasks become highly overlearned and “automatized”. When investigating cognitive control functions, however, researchers might want to avoid too much automaticity and overlearning of task-specific associations or stimulus–response rules, as these cognitive processes might alter or even substitute the cognitive control processes the researcher is interested in (see, e.g., Grange & Juvina, 2015; Scheil, 2016, for practice effects on *N *− 2 repetition costs; Davidson et al., 2003, for practice effects on the Stroop effect in young versus old adults; Strobach et al., 2014, for review).

## Experiment 1

In Experiment 1, we examined *N *− 2 repetition costs in task switching, which represent a measure of persisting inhibition of the most recent competing task. We also included an experimental variation of the CSI, which is the time for preparation of the upcoming task. We examined these effects on a group level, as well as their split-half and retest reliabilities on the level of interindividual differences. The group-level analysis also allowed us to explore whether *N *− 2 repetition costs are reduced with longer CSI.

### Method

#### Participant sample

96 participants were tested (72 female, 24 male; mean age 23.9 years, SD 3.9, range 18–36 years). They were students of Psychology (82 participants) or of other disciplines (14 participants), and received 12 Euros or partial course credits in return for participation. A sensitivity analysis conducted with the software G*Power 3.1.4 (Faul et al., 2007) revealed that a sample size of *N* = 96 allows for detecting effect sizes of *r* = 0.25 or larger (with a power of 0.80 and type I error rate of 0.05, one-tailed).

#### Number of trials per condition

*N *− 2 repetition costs and CSI effect were calculated on the basis of 960 trials in total (corresponding to 480 trials per session, and 240 trials per condition (ABA vs CBA; short vs long CSI) within each session, respectively).

#### Tasks, stimuli, and responses

The task-switching paradigm was similar to previous studies from our lab (e.g., Schuch & Grange, 2015, 2019; see Schuch et al., 2012, for a detailed description of the stimulus material). The stimuli were 40 different pictures of faces that had to be categorized as female or male (gender task), young or old (age task), or showing a happy or angry expression (emotion task). There were five pictures for each combination of gender, age, and emotional expression. The pictures were 10.6 cm by 14.1 cm in size, and were presented centrally on the computer screen. The tasks were indicated by the color of a frame that surrounded the facial pictures (frame size 11.0 cm by 14.5 cm, thickness of frame was 2 mm; blue frame color indicates the gender task, red indicates the age task, and yellow indicates the emotion task). The frame occurred 100 ms or 900 ms prior to the facial picture (manipulated blockwise; see below). Task cues and stimuli were presented on a 14-inch notebook screen (Dell Latitude D630), which was placed about 50 cm away from the participants. Participants used their left and right index fingers for responding, pressing the “x” and “,” keys on the notebook with QWERTZ layout (i.e., the response keys were located just above the left and right ends of the space bar, respectively). One half of participants responded to male, young, and happy faces by pressing the left key, and to female, old, and angry faces by pressing the key; for the other half of participants, the mapping was reversed.

#### Procedure

Every trial started with the presentation of a red, blue, or yellow frame for either 100 or 900 ms (depending on CSI condition), followed by the presentation of a picture inside the frame. Frame and picture stayed on the screen until the left or right response key was pressed. Then, the screen turned black for 1400 or 600 ms (depending on CSI condition). That is, the interval between the response in the previous trial and the stimulus in the current trial (response–stimulus interval, RSI) was constant across CSI conditions (1500 ms). If the wrong key was pressed, an error feedback occurred after 500 ms of blank screen and lasted for 1000 ms, after which the screen turned black again for another 900 or 100 ms (depending on CSI condition), leading to an RSI of 2500 ms after incorrect responses.

In the experimental blocks, the task cues and stimuli occurred in pseudorandom order with the following constraints. First, immediate task repetitions could not occur. Second, each task occurred equally often in each block. Third, there was a roughly equal number of *N *− 2 task repetitions and *N *− 2 task switches per block. Fourth, each stimulus was presented equally often in the context of each task. Fifth, the person presented in a particular trial n was never the same as the persons presented in trials *N *− 1 and *N *− 2. Sixth, half of the trials for each task required left-hand responses, and half required right-hand responses. Seventh, response repetitions and switches from trial *N *− 1 to trial *N*, and from trial *N *− 2 to trial *N*, occurred about equally often for each task.

Before the experiment, participants received instructions about the tasks. A reminder indicating the response mappings for all three tasks was placed below the screen for the whole experiment. Participants were told that the task-preparation interval would be short or long, alternating blockwise. For practice, participants performed eight short blocks of ten trials each (practice blocks 1 and 2: gender task; practice blocks 3 and 4: age task; practice blocks 5 and 6: emotion task; practice blocks 7 and 8: all three tasks intermixed; cue-stimulus interval [CSI] manipulated blockwise).

After practice, participants performed the first session, which consisted of eight experimental blocks of 60 trials each, with short and long CSI alternating blockwise. Whether participants started with short or long CSI was counterbalanced across participants (orthogonally to response mapping).^2^ The blocks were separated by short self-paced breaks. After the first session, there was a 10 min break, where participants performed an unrelated filler task.^3^ After the break, participants performed the second session. The second session was identical to the first, except that practice was shorter, consisting of two mixed-task blocks of ten trials each, one with short and one with long CSI.

#### Design and strategy of data analysis

Data analysis proceeded in two steps. In a first step, for the analysis of group-level effects, a 2 × 2 × 2 within-subjects design was applied, with the independent variables session (first vs. second), task sequence (ABA vs. CBA), and CSI (100 ms vs. 900 ms; blockwise manipulation). The dependent variables were RT and error rates. In a second step, we analyzed the reliability of *N *− 2 repetition costs and CSI effects, by calculating split-half reliability (as the correlation between odd-numbered and even-numbered trials) as well as retest reliability (as the correlation between first and second session). To allow for a direct comparison between the two reliability measures, split-half reliability was calculated on the complete data set (across both sessions, and without any correction for attenuation), so that both reliability measures are based on the same number of trials. For all statistical tests (ANOVAs and correlations), significance level was set to alpha = 0.05.

### Results

The first and second trial per block were excluded, because these could not be classified as ABA or CBA task sequence. Outliers were defined as trials with RT deviating more than three standard deviations from an individual’s overall mean RT, computed separately for first and second session, and were excluded as well (0.95% of the trials in the first session, 1.18% of the trials in the second session). Moreover, the two trials following an error were excluded, to eliminate influences of post-error processing. For RT analysis, error trials were excluded as well.

#### Experimental effects

**Fig. 1:**
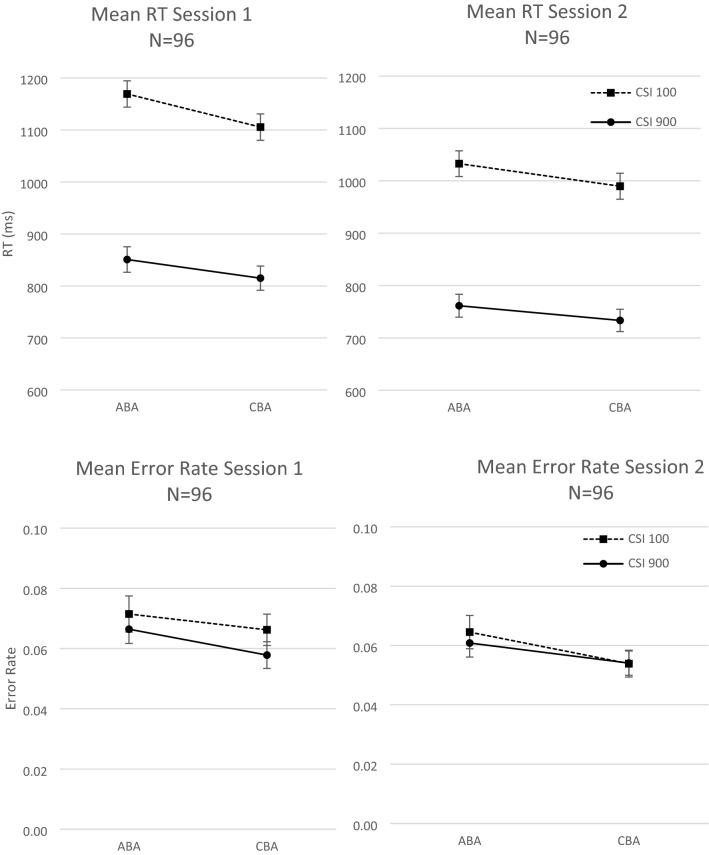
Experiment 1. Mean RT (in ms; upper row) and mean error rate (lower row) as a function of session (Sesison 1, Session 2), Task Sequence (ABA, CBA), and CSI (100 ms, 900 ms). Error bars indicate one standard error of mean

In a first step, we examined whether the standard experimental effects of *N *− 2 repetition costs and task-preparation time were obtained in both sessions (see Fig. 1). To this end, we performed ANOVAs with the independent variables session (first vs. second), task sequence (ABA vs. CBA), and CSI (100 ms vs. 900 ms).

The ANOVA on RT data revealed a significant main effect of CSI, *F*(1, 95) = 801.23, *p* < 0.01, *η*_*p*_^2^ = 0.89, and of Task Sequence, *F*(1, 95) = 125.59, *p* < 0.01, *η*_*p*_^2^ = 0.57, indicating standard task preparation effects (284 ms) and *N *− 2 task repetition costs (43 ms), respectively. There was also an interaction of task sequence and CSI, *F*(1, 47) = 11.33, *p* < 0.01, *η*_*p*_^2^ = 0.11, indicating larger *N *− 2 repetition costs with short than long CSI (53 ms vs. 32 ms). Moreover, there was a main effect of session, *F*(1, 95) = 60.10, *p* < 0.01, *η*_*p*_^2^ = 0.39, with shorter RTs in the second than first session (overall mean RT was 879 ms vs. 985 ms). Session interacted with CSI, *F*(1, 95) = 15.37, *p* < 0.01, *η*_*p*_^2^ = 0.14, indicating a smaller CSI effect in the second than first session (264 ms vs. 305 ms), and with task sequence, *F*(1, 95) = 5.35, *p* = 0.02, *η*_*p*_^2^ = 0.05, indicating smaller *N *− 2 task repetition costs in the second than first session (36 ms vs. 50 ms). The three-way interaction was not significant, *F*(1, 95) = 1.20, *p* = 0.28, *η*_*p*_^2^ = 0.01.

The respective ANOVA on error rates yielded a significant main effect of task sequence, *F*(1, 95) = 17.44, *p* < 0.01, *η*_*p*_^2^ = 0.16, confirming the *N *− 2 task repetition costs (0.8%). There was a marginally significant main effect of CSI, *F*(1, 95) = 3.69, *p* = 0.06, *η*_*p*_^2^ = 0.04, indicating a preparation effect in error rates of 0.4%. Furthermore, there was a main effect of session, *F*(1, 95) = 13.24, *p* < 0.01, *η*_*p*_^2^ = 0.12, with fewer errors in the second than first session (5.8% vs. 6.5%). No other effects reached significance, all *F*(1, 95)s < 2.09.

#### Reliability analyses

We first report reliability analyses of *N *− 2 repetition costs, followed by reliability analyses of the task-preparation effect (i.e., CSI effect). For both effects, we start with split-half reliability and then report retest reliability. Split-half reliability was calculated by separating the combined data of both sessions into two halves (odd trials versus even trials), and computing the Pearson correlation coefficient between the two halves. Retest reliability was computed as the Pearson correlation coefficient between the first and second session. Note that in this way, the calculation of split-half and retest reliability is based on the same number of trials, and hence the reliability scores can be directly compared.

In addition, we also computed split-half reliability on the basis of random splits of the data. While it is a common procedure to compute split-half reliability by dividing the data into odd- and even-numbered trials, this is just one out of many ways of splitting the data into halves. Following Kowalczyk and Grange (2017) and Congdon et al. (2012), we also performed a bootstrapping analysis where we randomly split the data into two halves and computed split-half reliability for each random split, repeating this procedure for 1000 times. This allowed us to get an estimate of the average split-half reliability and its variability.^4^

**Fig. 2:**
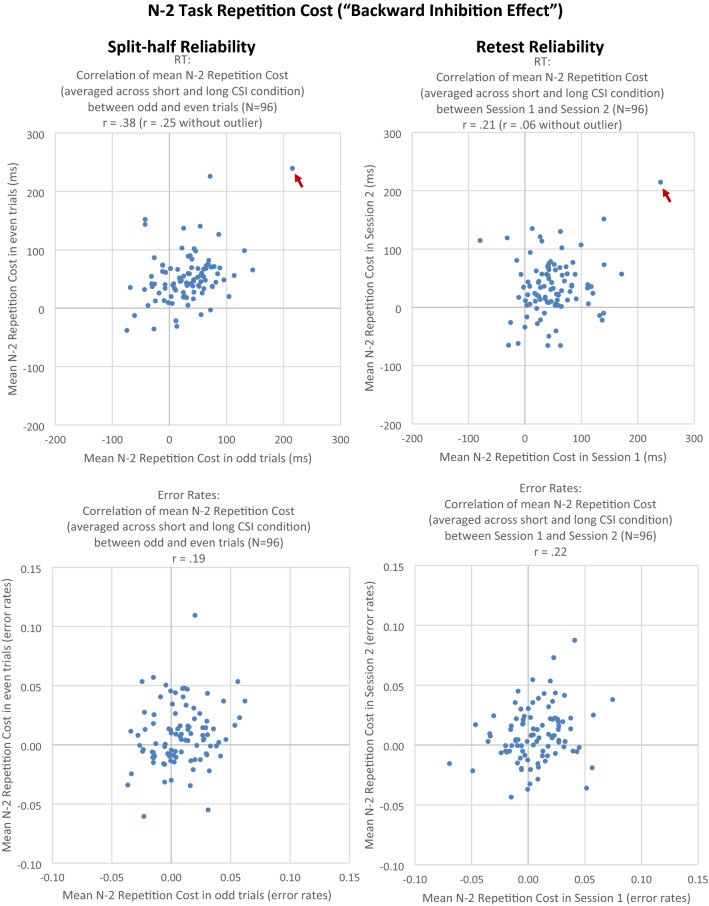
Experiment 1. Left column: correlation of *N *− 2 repetition cost between odd and even trials (split-half reliability). Right column: correlation of *N *− 2 repetition cost between Session 1 and Session 2 (retest reliability). Upper row: RT (in ms). Lower row: error rate. One dot represents one individual subject. Data points indicated by a red arrow were treated as outliers. The RT outliers in the plots for split-half and retest reliability stem from the same participant

For *N *− *2 repetition costs,* the odd–even *split-half reliability* (averaged across CSI conditions) was *r* = 0.38, *t*(94) = 4.03, *p* < 0.01; when one outlying data point was excluded, the correlation dropped to *r* = 0.25, *t*(93) = 2.49, *p* = 0.01 (see Fig. 2). Because we found that *N *− 2 repetition costs significantly interacted with CSI in the ANOVA, we also computed odd–even split-half reliability of *N *− 2 repetition costs separately for the different CSI conditions. For the short CSI condition, split-half reliability of *N *− 2 repetition costs was *r* = 0.22, *t*(94) = 2.19, *p* = 0.04, for *N *− 2 repetition costs in the short CSI condition, and *r* = 0.27, *t*(94) = 2.72, *p* = 0.01, for *N *− 2 repetition costs in the long CSI condition. Note that when computing split-half reliabilities separately for the short and long CSI condition, the number of trials is only half the size, which attenuates the observed correlations; to compare these correlations with the overall split-half reliability, we, therefore, applied the Spearman–Brown formula. The corrected split-half reliabilities amount to *r*_c_ = 0.36 for *N *− 2 repetition costs in the short CSI condition, and *r*_c_ = 0.42 for *N *− 2 repetition costs in the long CSI condition.

In error data, the odd–even split-half reliability of *N *− 2 repetition costs (averaged across CSI conditions) was *r* = 0.19, *t*(94) = 1.83, *p* = 0.07. When computed separately for the different CSI conditions, the split-half reliability was *r* = 0.24, *t*(94) = 2.40, *p* = 0.02, *r*_c_ = 0.39, for the short CSI condition, and *r* = 0.00, *t*(94) < 1, *r*_c_ = 0.01, for the long CSI condition.

Next, we calculated *retest reliability* of *N *− 2 repetition costs. In RT data, the correlation of *N *− 2 repetition costs between sessions was *r* = 0.21, *t*(94) = 2.08, *p* = 0.04; when one outlying data point was excluded, the correlation dropped to *r* = 0.06, *t*(93) < 1 (see Fig. 2). When computed separately for the short and long CSI condition, the correlation was *r* = 0.23, *t*(94) = 2.34, *p* = 0.02, *r*_c_ = 0.38, for the short CSI condition, and *r* = 0.09, *t*(94) < 1, *r*_c_ = 0.16, for the long CSI condition. In error data, the correlation of *N *− 2 repetition costs (averaged across CSI conditions) between sessions was *r* = 0.22, *t*(94) = 2.22, *p* = 0.03. When computed separately for the different CSI conditions, the correlation was *r* = 0.25, *t*(94) = 2.52, *p* = 0.01, *r*_c_ = 0.40, for the short CSI condition, and *r* = 0.05, *t*(94) < 1, *r*_c_ = 0.09, for the long CSI condition.

**Fig. 3:**
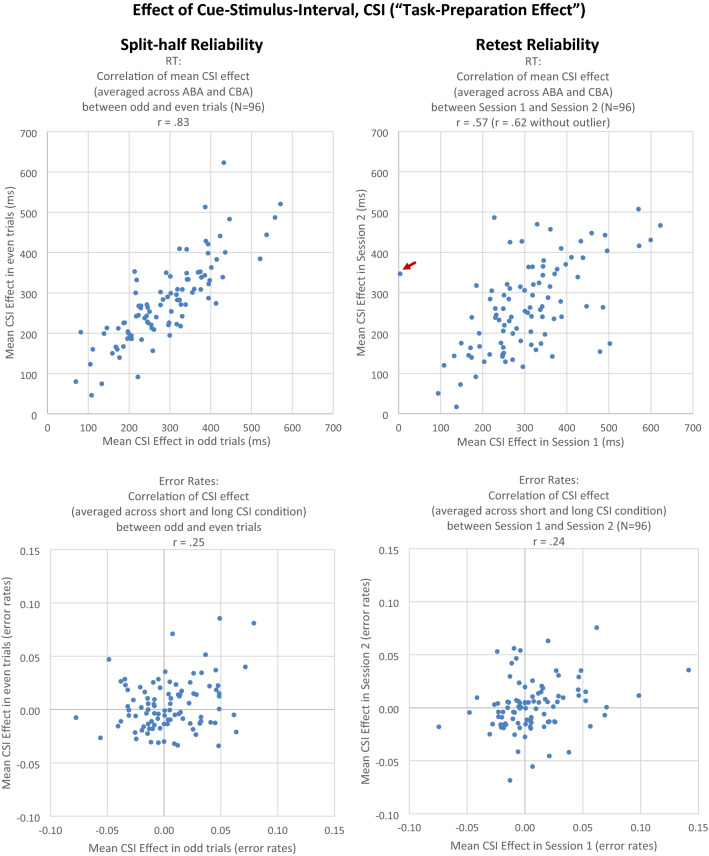
Experiment 1. Left column: Correlation of CSI effect between odd and even trials (split-half reliability). Right column: correlation of CSI effect between Session 1 and Session 2 (retest reliability). Upper row: RT (in ms). Lower row: error rate. One dot represents one individual subject. Data points indicated by a red arrow were treated as outliers

For the *CSI effect*, we first calculated odd–even *split-half reliability* for the RT data and found a correlation of the CSI effect (computed as mean RT in short CSI minus mean RT in long CSI, averaged across ABA and CBA) in odd versus even trials of *r* = 0.83, *t*(94) = 9.99, *p* < 0.01 (see Fig. 3). In error data, the odd–even correlation (averaged across ABA and CBA) was *r* = 0.25, *t*(94) = 2.52, *p* = 0.02.

Next, we calculated *retest reliability* of the CSI effect. In RT data, the correlation of the CSI effect (averaged across ABA and CBA) between sessions was *r* = 0.57, *t*(94) = 6.81, *p* < 0.01; when one outlying data point was excluded, the correlation was *r* = 0.62, *t*(94) = 7.71, *p* < 0.01 (see Fig. 3). In the error data, the correlation of the CSI effect (averaged across ABA and CBA) between sessions was *r* = 0.24, *t*(94) = 2.43, *p* = 0.02.

To summarize, for *N *− 2 repetition costs, we found moderate to low split-half and retest reliabilities. For the CSI effect we found a good split-half reliability in RT; the retest reliability in RT was somewhat smaller but still acceptable.

#### Comparison of correlation coefficients

We also checked whether retest reliabilities were statistically smaller than odd–even split-half reliabilities, using the “cocor” web interface (http://comparingcorrelations.org; Diedenhofen & Musch, 2015). The results showed that for *N *− 2 repetition costs, retest reliability was significantly smaller than split-half reliability: When comparing the correlations based on *N* = 96 (i.e., with the outlier included), Pearson and Filon’s *z* = 1.84, *p* = 0.03, one-tailed. When comparing the correlations based on *N* = 95 (i.e., with the outlier excluded), Pearson and Filon’s *z* = 1.92, *p* = 0.027, one-tailed; the other statistical tests available with this software yielded very similar results). Likewise, for the CSI effect, retest reliability was significantly smaller than split-half reliability; for *N* = 96 (i.e., with the outlier included): *z* = 4.36, *p* = 0.00, one-tailed; for *N* = 95 (i.e., with the outlier excluded): *z* = 3.84, *p* = 0.00, one-tailed). The descriptive statistics of the distributions of *N *− 2 repetition costs and task-preparation effects are summarized in Table 1.

**Table 1:** Descriptive statistics of the distributions of N-2 repetition costs and task-preparation effect (Experiment 1) and congruency effect and conflict-adaptation effect (Experiment 2)

	Mean		Standard deviation	Skew		Kurtosis
***Experiment 1 (N = 96)***						
*N − 2 repetition costs in RT (ms)*						
Odd trials	32		47	0.46		1.72
Even trials	53		45	1.40		4.59
Session 1 trials	50		48	0.84		2.39
Session 2 trials	36		49	0.53		1.43
*N − 2 repetition costs in error rates*						
Odd trials	0.008		0.022	0.19		− 0.36
Even trials	0.008		0.026	0.56		1.91
Session 1 trials	0.007		0.024	− 0.16		0.77
Session 2 trials	0.009		0.023	0.51		1.05
*Task-preparation effect in RT (ms)*						
Odd trials	290		104	0.32		0.03
Even trials	279		102	0.52		0.84
Session 1 trials	305		112	0.47		0.71
Session 2 trials	264		110	0.22		− 0.66
*Task-preparation effect in error rates*						
Odd trials	0.005		0.030	0.16		− 0.041
Even trials	0.004		0.024	0.99		1.50
Session 1 trials	0.007		0.031	1.26		4.00
Session 2 trials	0.002		0.024	0.34		1.24
***Experiment 2 (N = 48)***						
*Congruency effect in RT (ms)*						
Odd trials		34	19	0.27	0.49	
Even trials		37	20	1.18	2.80	
Session 1 trials		40	24	1.23	4.22	
Session 2 trials		30	17	0.15	0.51	
*Congruency effect in error rates*						
Odd trials		0.036	0.037	0.92	1.41	
Even trials		0.034	0.028	0.07	− 0.76	
Session 1 trials		0.035	0.033	0.21	− 0.55	
Session 2 trials		0.034	0.035	1.04	1.17	
*Conflict adaptation effect in RT (ms)*						
Odd trials		17	31	1.40	3.80	
Even trials		-3	28	0.16	0.16	
Session 1 trials		7	32	0.26	0.41	
Session 2 trials		8	33	1.16	6.45	
*Conflict adaptation effect in error rates*						
Odd trials		0.011	0.055	0.04	2.23	
Even trials		0.022	0.043	0.14	− 0.59	
Session 1 trials		0.023	0.050	− 0.17	2.09	
Session 2 trials		0.008	0.047	0.61	0.47	

#### Bootstrapping analysis of split-half reliability

Since splitting the data into odd- and even-numbered trials is just one possibility of dividing the data into two halves, we additionally performed a bootstrapping analysis, where we performed 1000 random splits of the data and computed split-half reliability for each random split (see Congdon et al., 2012; Kowalczyk & Grange, 2017, for a similar approach). We did this separately for *N *− 2 repetition costs (averaged across CSI conditions, *N* = 96) and for the CSI effect (averaged across ABA and CBA sequences, *N* = 96); for each effect, we computed the bootstrapped split-half reliabilities for the combined data of both sessions, as well as separately for each session. In Table 2, we report the median, and 95% range around the median, of the bootstrapped split-half reliabilities.

**Table 2:** Split-half reliabilities of N-2 repetition costs and task-preparation effect (Experiment 1), and congruency effect and conflict-adaptation effect (Experiment 2), computed separately for Session 1, Session 2, and pooled across Sessions 1 and 2

	Session 1	Session 2	Pooled across Sessions 1 and 2
***Experiment 1 (N = 96)***			
*N − 2 repetition cost in RT*			
Odd–even	*r* = 0.25, *r*_c_ = 0.40	*r* = 0.22, *r*_c_ = 0.36	*r* = 0.38
Bootstrapped	*r*_median_ = 0.33, *r*_c_ = 0.50 95% range [0.11; 0.55]	*r*_median_ = 0.16, *r*_c_ = 0.28 95% range [− 0.11; 0.38]	*r*_median_ = 0.47 95% range [0.24; 0.64]
*N − 2 repetition cost in error rates*			
Odd–even	*r* = − 0.16	*r* = − 0.05	*r* = 0.19
Bootstrapped	*r*_median_ = − 0.04 95% range [− 0.20; 0.13]	*r*_median_ = − 0.03 95% range [− 0.21; 0.15]	*r*_median_ = 0.06 95% range [− 0.12; 0.23]
*Task-preparation effect in RT*			
Odd–even	*r* = 0.71, r_c_ = 0.83	*r* = 0.74, r_c_ = 0.85	*r* = 0.83
Bootstrapped	*r*_median_ = 0.90, *r*_c_ = 0.95 95% range [0.84; 0.94]	*r*_median_ = 0.90, *r*_c_ = 0.95 95% range [0.82; 0.94]	*r*_median_ = 0.94 95% range [0.91; 0.96]
*Task-preparation effect in error rates*			
Odd–even	*r* = 0.17, *r*_c_ = 0.29	*r* = 0.15, *r*_c_ = 0.26	*r* = 0.25
Bootstrapped	*r*_median_ = 0.19, *r*_c_ = 0.32 95% range [0.03; 0.34]	*r*_median_ = 0.04, *r*_c_ = 0.08 95% range [− 0.13; 0.22]	*r*_median_ = 0.21 95% range [0.03; 0.36]
***Experiment 2 (N = 48)***			
*Congruency effect in RT*			
Odd–even	*r* = 0.44, *r*_c_ = 0.61	*r* = − 0.03	*r* = 0.34
Bootstrapped	*r*_median_ = 0.26, *r*_c_ = 0.41 95% range [− 0.04; 0.51]	*r*_median_ = 0.07 95% range [− 0.29; 0.36]	*r*_median_ = 0.27 95% range [− 0.04; 0.51]
*Congruency effect in error rates*			
Odd–even	*r* = 0.33, *r*_c_ = 0.50	*r* = 0.43, *r*_c_ = 0.60	*r* = 0.53
Bootstrapped	*r*_median_ = 0.29, *r*_c_ = 0.45 95% range [0.08; 0.50]	*r*_median_ = 0.39, *r*_c_ = 0.56 95% range [0.20; 0.58]	*r*_median_ = 0.51 95% range [0.32; 0.66]
*Conflict adaptation effect in RT*			
Odd–even	*r* = − 0.28	*r* = − 0.36	*r* = − 0.01
Bootstrapped	*r*_median_ = − 0.15 95% range [− 0.44; 0.15]	*r*_median_ = 0.00 95% range [− 0.34; 0.33]	*r*_median_ = − 0.17 95% range [*− *0.47; 0.15]
*Conflict adaptation effect in error rates*			
Odd–even	*r* = 0.34, *r*_c_ = 0.51	*r* = − 0.08	*r* = 0.20
Bootstrapped	*r*_median_ = 0.04, *r*_c_ = 0.08 95% range [− 0.20; 0.28]	*r*_median_ = 0.01 95% range [− 0.19; 0.24]	*r*_median_ = 0.12 95% range [− 0.11; 0.34]

Odd–even: Split-half reliability computed by splitting the data into odd and even trials. Bootstrapped: Split-half reliability computed by randomly splitting the data into two halves, with 1000 iterations. *r*_median_ indicates the median of the bootstrapped correlations; 95% range indicates the 2.5th and 97.5th percentile of the distribution of correlations. *r*: uncorrected correlation. *r*_c_: Spearman-Brown corrected correlation. The Spearman–Brown correction was applied to the odd–even split-half reliabilities that were computed separately for Session 1 and Session 2, but only if these reliabilities were larger than zero. For negative split-half reliability values, it was assumed that reliability is zero

The median bootstrapped reliability of *N *− 2 repetition costs for the combined data of both sessions was slightly higher than the respective odd–even split-half reliability in RT (*r*_median_ = 0.47; 95% range from *r* = 0.24 to *r* = 0.64; versus *r*_odd–even_ = 0.38) and close to zero in error rates (*r*_median_ = 0.06; 95% range from *r* = − 0.12 to *r* = 0.23). For the CSI effect, the median bootstrapped reliability in RT was remarkably high, with 95% of the estimates above *r* = 0.90 (*r*_median_ = 0.94; 95% range from *r* = 0.91 to *r* = 0.96; versus *r*_odd–even_ = 0.83); in error rates, it was low (*r*_median_ = 0.21; 95% range from *r* = 0.03 to *r* = 0.36). Overall, the bootstrapped reliabilities showed a similar data pattern as the odd–even reliabilities: split-half reliability of the CSI effect in RT was very good (only in RT, not in error rates), and split-half reliability of *N *− 2 repetition costs was moderate to poor.

When looking at the sessionwise reliability estimates, as expected, these were lower than the reliabilities estimated from the combined data of two sessions, due to the smaller trial numbers. When applying the Spearman-Brown correction to the sessionwise reliabilities, the reliability estimates were comparable to the estimates of the combined data (see Table 2).

### Discussion

We will first discus the group-level effects as obtained in the ANOVAs, and then turn to the reliability analyses. This experiment showed very robust main effects of *N *− 2 task repetition and of CSI. That is, there were overall quite sizeable *N *− 2 task repetition costs of about 40 ms, and the general preparation effect was also very substantial and showed a RT benefit of 284 ms with long CSI relative to short CSI. These two effects replicate established basic effects. Moreover, we observed that *N *− 2 repetition costs became smaller with longer preparation time.

Effects of task-preparation time are of high theoretical relevance in task switching research. The finding of better performance with long than short CSI (i.e., main effect of CSI) is probably one of the most robust findings in task-switching research (see Kiesel et al., 2010), and can possibly be related to activation of the cued task representation.

The finding of smaller *N *− 2 repetition costs with long than short CSI confirms findings from Gade and Koch (2014, Experiments 1 and 2), Scheil and Kleinsorge (2014, Experiment 2), and Schuch and Grange (2019, Experiment 2), and extends them to conditions with blocked CSIs. Notably, other previous studies did not find any modulation of N-2 repetition costs by CSI, despite substantial general preparation effects (i.e., main effect of CSI but no interaction with *N *− 2 repetition costs; e.g., Lawo et al., 2012; Mayr & Keele, 2000; Prior, 2012; Schuch & Koch, 2003). Given that the effect size of the modulation of *N *− 2 repetition costs by CSI was rather small in the present study (*η*_*p*_^2^ = 0.11 for the interaction of CSI and *N *− 2 repetition costs), it is possible that larger sample sizes are needed to observe this effect (e.g., sample size was *N* = 96 in the present study, , as opposed to Ns between 16 and 24 in the studies that did not find the modulation).

The finding of smaller *N *− 2 repetition costs with long than short CSI suggests that part of the persisting inhibition of a previously abandoned task set can be overcome when preparation time for the upcoming task is longer. This finding resembles the finding of smaller task-switch costs (i.e., smaller difference between task-switch and task-repetition trials) with long than short CSI, which has been interpreted as a marker of “endogenous” reconfiguration of task set, and as such a hallmark of endogenous cognitive control (e.g., Monsell, 2003; see Kiesel et al., 2010; Vandierendonck et al., 2010, for discussions).

Moreover, the design with two sessions that were separated by a short break allowed us to examine practice effects. We observed that performance improved generally (i.e., RT decreased) from first to second session, and both CSI effect and *N *− 2 repetition costs became significantly smaller in the second session. The latter finding is in line with previous studies observing a reduction of *N *− 2 repetition costs with practice (Grange & Juvina, 2015; Scheil, 2016). Grange and Juvina (2015) tested a small number of participants (*N* = 9) who practised extensively (over five sessions with more than 1200 trials each); here, we observed practice effects in a sample more than 10 times larger (*N* = 96), where participants received relatively little practice (only two sessions with 480 experimental trials each).

The focus of the present study was on the reliability of the cognitive-control measures. Regarding *N *− 2 repetition costs, we observed an odd–even split-half reliability of *r* = 0.38 (*r* = 0.25 when excluding one outlying data point), and bootstrapped split-half reliability of *r*_median_ = 0.47, which is at the lower end of the split-half reliabilites reported in previous studies, ranging between *r* ≈ 0.30 and *r* ≈ 0.60 (Kowalczyk & Grange, 2017; Pettigrew & Martin, 2016; Rey-Mermet et al., 2018). Note that such reliability scores depend on the number of experimental trials that provide the basis for the correlation, and that most earlier studies reported corrected split-half reliability scores using the Spearman–Brown formula (which corrects for halving trial numbers when splitting them into odd and even trials). For instance, Kowalczyk and Grange (2017) reported corrected (*r* ≈ 0.50) and uncorrected (*r* ≈ 0.33) split-half reliability from *N *− 2 repetition cost paradigms comprising 480 trials in total. For a direct comparison, it is warranted to compare their corrected scores (which are based on 480 trials in total) to the present uncorrected scores (which are based on 960 trials in total); we thus observed a somewhat lower split-half reliability of *N *− 2 repetition costs (*r* ≈ 0.30 to *r* ≈ 0.45) compared to the study by Kowalczyk and Grange (2017), who observed *r* ≈ 0.50.

An important new result of the present experiment is that we also calculated retest reliability as the correlation of the respective effect in the first session with that in the second session. Even though sessions were separated by only 10 min, the retest reliability of *N *− 2 repetition costs was low, with *r* = 0.21 (*r* = 0.06 when excluding one outlying data point). Retest reliability of *N *− 2 repetition costs was significantly lower than split-half reliability, even though the same participants performed the identical experiment in Session 1 and 2 on the same day, and the sessions were only separated by 10 min. The only systematic difference between Session 1 and Session 2 are practice effects, with somewhat smaller *N *− 2 repetition costs in the second than first session.

The low retest reliability of *N *− 2 repetition costs suggests that this measure is not suitable as a measure of a stable, trait-like cognitive ability of task inhibition, confirming recent doubts about the stability of inhibition as a psychometric construct in studies of interindividual cognitive differences (see also Kowalczyk & Grange, 2017; Rey-Mermet et al., 2018). We will return to this issue in the General Discussion.

Other than *N *− 2 repetition costs, the CSI effect yielded a good split-half reliability (odd–even split-half reliability of *r* = 0.83, again, the uncorrected score is reported; bootstrapped split-half reliability of *r*_median_ = 0.94), and reasonable retest reliability (*r* = 0.62 when excluding one outlying data point). The split-half reliability estimates are well above the value of *r* = 0.70, which is often taken as a lower limit for acceptable split-half reliability (Cronbach, 1951). The retest reliability score suggests that the CSI effect is suitable as a measure of interindividual differences in cue-based task preparation in task switching.

## Experiment 2

In Experiment 2, we extended our exploration of split-half and retest-reliability to two other prominent cognitive control measures, both of which can be measured in single-task paradigms: a Stroop-like face-name interference effect, and the conflict adaptation effect. Face-name interference effects are popular in the imaging literature (because faces and names activate clearly distinguishable brain areas; e.g., Egner & Hirsch, 2005); the conflict-adaptation effect has inspired a massive research endeavor to better understand the underlying mechanisms in terms of conflict-triggered adjustments of cognitive control (e.g., Egner, 2008; Schuch et al., 2019).

### Method

#### Participants

48 new participants were tested (43 female, 5 male).^5^ Their mean age was 20.7 years (SD 2.6, range 18–32 years). All were students of Psychology. They received partial course credits in return for participation. A sensitivity analysis conducted with the software G*Power 3.1.4 (Faul et al., 2007) revealed that this sample size allows for detecting effect sizes of *r* = 0.34 or larger (with a power of 0.80 and type I error rate of 0.05, one-tailed).

#### Number of trials per condition

Congruency effects and sequential congruency effects were calculated on the basis of 800 trials in total (corresponding to 400 trials per session, with 200 congruent and 200 incongruent trials within each session).

#### Task, stimuli and responses

We used a face-name Stroop-like paradigm that was similar to previous studies from our lab (Schuch & Koch, 2015; Schuch et al., 2017). Pictures of female and male faces were presented, with a female or male name superimposed onto each face. Participants had to classify the gender of the name while ignoring the gender of the face. Half of the stimuli were congruent (i.e., name and face were of same gender), half incongruent (name and face had different genders). The stimulus set consisted of 20 different faces (10 female and 10 male), and 20 different names (10 female and 10 male), resulting in 400 possible face-name combinations.

The pictures were 10.6 cm in width and 14.1 cm in height (i.e., same picture size as in Experiment 1; for further details of the pictures, see Schuch et al., 2012). Across each face, a name was printed centrally in bright blue color (font size 48). The names were displayed over the noses but not in the region of the eyes or mouth. In half of the trials, the gender of name and face corresponded (congruent trials); in the other half, they did not correspond (incongruent trials). 20 common German first names were used (the male names were Anton, Christian, Frank, Kevin, Leon, Markus, Niklas, Sebastian, Thorsten, Ulf; the female names were Anke, Carolin, Frauke, Kerstin, Lena, Meike, Nathalie, Stefanie, Tamara, Ulrike; cf. Schuch & Koch, 2015; Schuch et al., 2017). The stimuli were presented on the same notebook as in Experiment 1.

The participants’ task was to categorize the name as male or female while ignoring the gender of the face. Half of the participants in each group pressed a left key for male names and right key for female names; for the other half the mapping was reversed. The same keys were used as in Experiment 1, and participants responded with the left and right index fingers.

#### Procedure

Each trial started with simultaneous presentation of a face and name. These stayed on the screen until the left or right response key was pressed. After correct responses, the computer screen turned black for 500 ms before the next face and name occurred. After a wrong key press, the screen turned black for 500 ms, then an error message was displayed for 1000 ms.

The experimental procedure was analogous to that in Experiment 1. Participants received instructions about the task, and a reminder indicating the response mapping was placed below the screen for the whole experiment. Then, participants completed four short practice blocks of ten trials each, followed by four experimental blocks with 100 trials each. During the 400 experimental trials, every possible combination of face and name was displayed once. The sequence of trials was randomized within the individual blocks, with the constraints that each block consisted of an equal number of congruent and incongruent trials, and each face and each name occurred at least once per block. As in Experiment 1, a 10-min break occurred between first and second session, during which participants performed an unrelated filler task (i.e., either a crossword puzzle or a mindfulness meditation).^6^ For Session 2, the experimental paradigm was identical to that in Session 1, except that practice was shorter, consisting of one short block of ten trials.

#### Design and strategy of data analysis

As with Experiment 1, data analysis proceeded in two steps. First, for the analysis of group-level effects, a 2 × 2 × 2 within-subjects design was applied, with the independent variables session (first vs. second), congruency (congruent vs. incongruent), and previous congruency (previous congruent vs. previous incongruent).The dependent variables were RT and error rates. Second, we analyzed the reliability of congruency effects and sequential congruency effects, by calculating split-half reliability (as the correlation between odd and even trials) and retest reliability (as the correlation between first and second session). As with Experiment 1, split-half reliability was calculated on the complete data set (including Session 1 and 2), to allow for a direct comparison between the two reliability measures. Significance level was set to alpha = 0.05.

### Results

The first trial of each block was excluded. Outliers were defined as trials with RT deviating more than three standard deviations from an individual’s overall mean RT, computed separately for Session 1 and 2 and were excluded as well (1.85% of the trials in Session 1, 1.86% in Session 2). To minimize episodic retrieval effects that might compromise the measurement of sequential congruency effects (Duthoo et al., 2014; Egner, 2007, 2017; Hommel et al., 2004; Mayr & Awh, 2009; Mayr et al., 2003; Whitehead et al., 2019), trials with repetitions of pictures and repetitions of names from trial *N *− 1 to trial *N* were excluded (9.25% of the trials). Moreover, trials immediately following an error were excluded to eliminate influences of post-error processing. For RT analysis, error trials were excluded as well.

#### Experimental effects

**Fig. 4:**
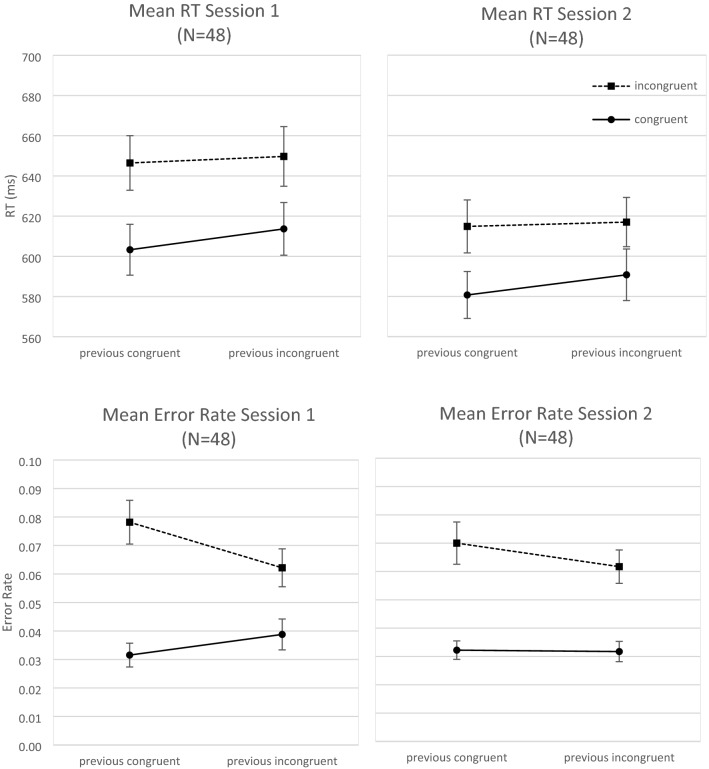
Experiment 2. Mean RT (in ms; upper row) and mean error rate (lower row) as a function of session (Session 1, Session 2), congruency (congruent, incongruent), and previous congruency (previous congruent, previous incongruent). Error bars indicate 1 standard error of mean

The ANOVA on RT data revealed a significant main effect of congruency, *F*(1, 47) = 245.73, *p* < 0.01, *η*_*p*_^2^ = 0.84, indicating shorter RTs for congruent than incongruent trials, as well as a main effect of previous congruency, *F*(1, 47) = 13.75, *p* < 0.01, *η*_*p*_^2^ = 0.23, indicating shorter RTs after congruent than after incongruent trials. There was also an interaction of congruency and previous congruency, *F*(1, 47) = 6.78, *p* = 0.01, *η*_*p*_^2^ = 0.13, indicating a larger congruency effect following congruent trials than following incongruent trials (39 ms vs. 31 ms) and thus a sequential congruency effect (see Fig. 4). Moreover, there was a main effect of session, *F*(1, 47) = 28.16, *p* < 0.01, *η*_*p*_^2^ = 0.38, with shorter RTs in Session 2 than in Session 1 (601 ms vs. 628 ms), and an interaction of session and congruency, *F*(1, 47) = 6.11, *p* = 0.02, *η*_*p*_^2^ = 0.12, indicating smaller congruency effect in Session 2 than in Session 1 (30 ms vs. 40 ms). No other effects were significant, *F*s < 1.

In error data, the ANOVA yielded a significant main effect of congruency, *F*(1, 47) = 69.60, *p* < 0.01, *η*_*p*_^2^ = 0.60, indicating fewer errors in congruent than incongruent trials, and a trend for a main effect of previous congruency, *F*(1, 47) = 3.14, *p* = 0.08, *η*_*p*_^2^ = 0.06. There was also an interaction of congruency and previous congruency, *F*(1, 47) = 8.26, *p* = 0.01, *η*_*p*_^2^ = 0.15, indicating a sequential congruency effect (with the congruency effect amounting to 4.2% after congruent trials and 2.7% after incongruent trials), as well as a trend for a three-way interaction with session, *F*(1, 47) = 2.94, *p* = 0.09, *η*_*p*_^2^ = 0.06, indicating a marginally larger sequential congruency effect in Session 1 than Session 2. There was no significant main effect of session, *F*(1, 47) = 2.29, *p* = 0.14, *η*_*p*_^2^ = 0.05, and no other effects, *F*s < 1.

Hence, the data pattern was similar in RT and error data. There were significant congruency effects and sequential congruency effects in both error data and RT data (see Fig. 4).

#### Reliability analyses

**Fig. 5:**
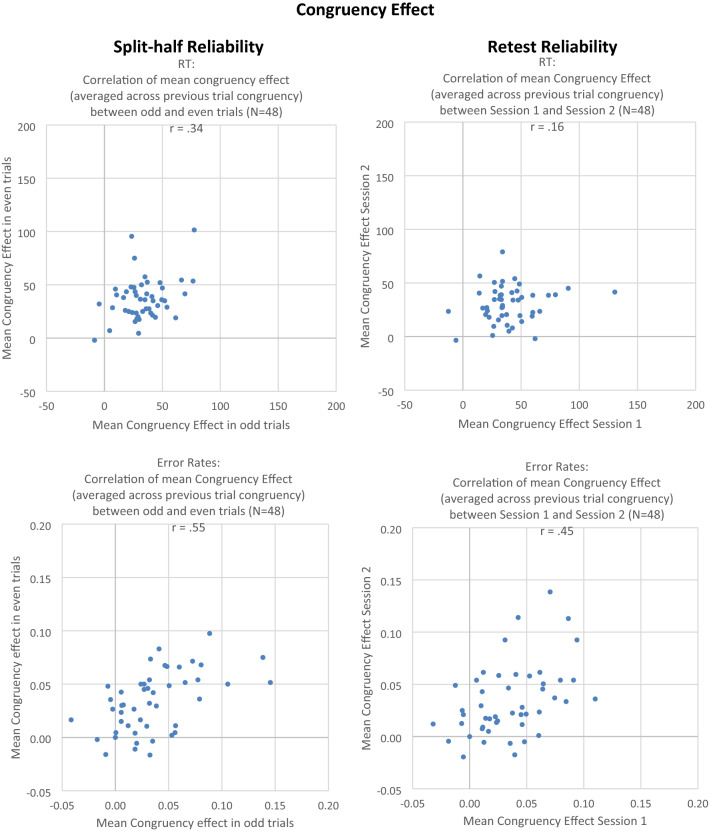
Experiment 2. Left column: correlation of congruency effect between odd and even trials (split-half reliability). Right column: correlation of congruency effect between Session 1 and Session 2 (retest reliability). Upper row: RT (in ms). Lower row: error rate. One dot represents one individual subject

**Fig. 6:**
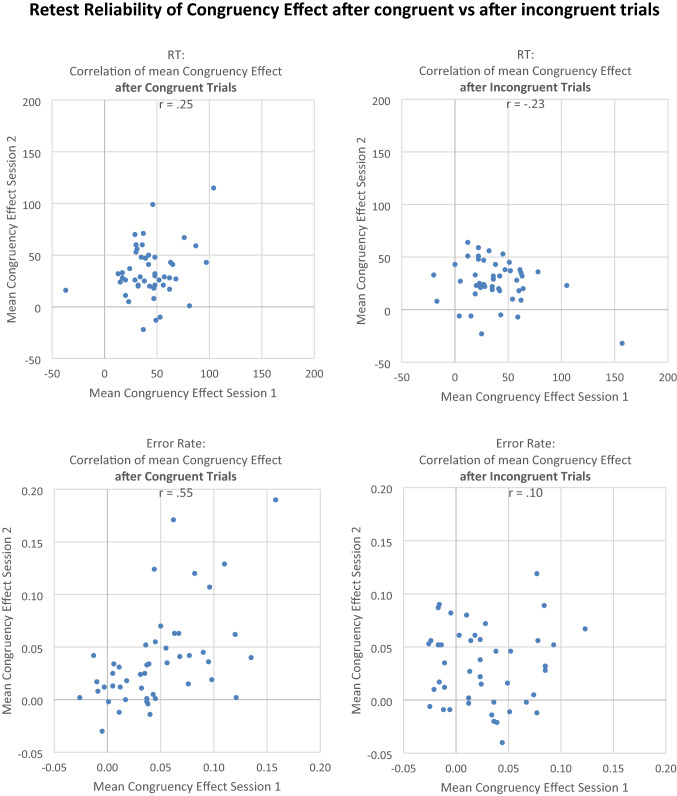
Experiment 2. Correlation of congruency effect between Session 1 and Session 2. Left: congruency effect after previous congruent trials. Right: congruency effect after previous incongruent trials. Upper row: RT (in ms), lower row: error rate. One dot represents one individual subject

**Fig. 7:**
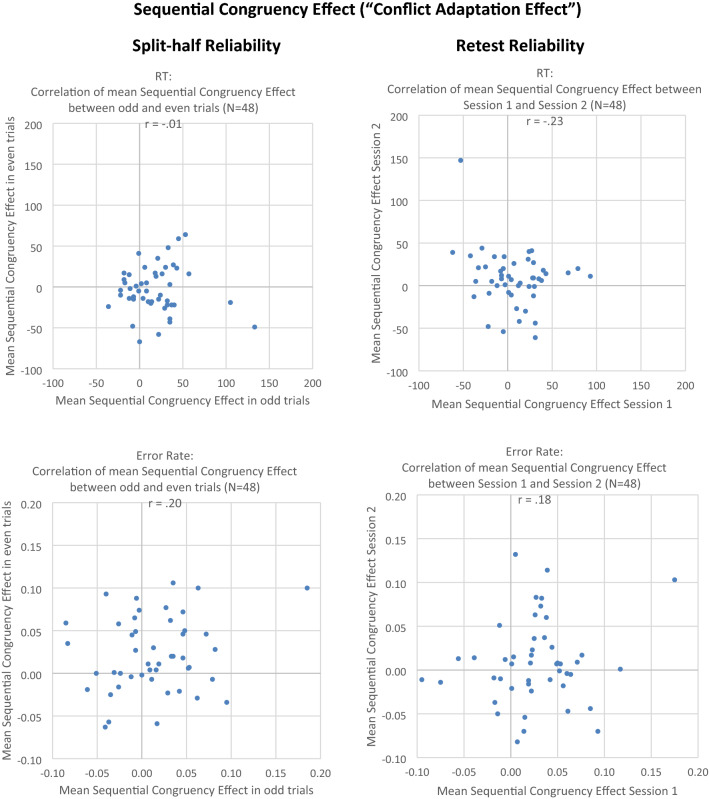
Experiment 2. Left column: correlation of sequential congruency effect between odd and even trials (split-half reliability). Right column: correlation of sequential congruency effect between Session 1 and Session 2 (retest reliability). Upper row: RT (in ms), lower row: error rate. One dot represents one individual subject

See Figs. 5, 6, and 7. For calculating *split-half reliability*, the combined data from both sessions were divided into two subsets (odd trials versus even trials), and the correlation of the effect between the two subsets was computed. In RT data, the correlation of the congruency effect (averaged across previous congruency) was *r* = 0.34, *t*(46) < 2.46, *p* = 0.02. The obtained Stroop-like effect, as measured by the main effect of congruency in the ANOVA, was very large as an experimental effect (*η*_*p*_^2^ = 0.84). However, research on the sequential congruency effects tells us that the congruency effect should be observed primarily after congruent trials, and this is what we found, too (i.e., Stroop effect of 39 ms vs. 31 ms in RT data; Stroop effect of 4.2% vs. 2.7% in Error Data). In fact, when the Stroop effect was analyzed separately for trials preceded by congruent versus incongruent stimuli, split-half reliability for the Stroop effect in trials preceded by a previous congruent stimulus was *r* = 0.52 (*r*_c_ = 0.69), *t*(46) = 4.16, *p* = 0.01. In contrast, split-half reliability for the Stroop effect in trials preceded by a previous incongruent stimulus was *r* =  − 0.02, *t*(46) < 1, thus showing a reliability of virtually zero. Consistent with this finding, for the sequential congruency effect, which represents the difference between the Stroop effects preceded by congruent vs. incongruent stimuli, the correlation was *r* =  − 0.01, *t*(46) < 1.

In error data, the split-half reliability of the congruency effect (averaged across previous congruency) was *r* = 0.53, *t*(46) = 4.2, *p* < 0.01. For trials preceded by a previous congruent stimulus, the correlation was *r* = 0.45 (*r*_c_ = 0.62), *t*(46) = 3.40, *p* < 0.01; for trials preceded by a previous incongruent stimulus, the correlation was *r* = 0.34 (*r*_c_ = 0.51), *t*(46) = 2.45, *p* = 0.02. For the sequential congruency effect, the correlation was *r* = 0.20, *t*(46) = 1.35, *p* = 0.19.

For *retest reliability*, in the RT data, the correlation of the congruency effect (computed as mean RT in incongruent minus mean RT in congruent trials, averaged across previous congruent and previous incongruent trials) between Session 1 and 2 was *r* = 0.16, *t*(46) = 1.10, *p* = 0.28. Based on the assumption that the congruency effect should be found primarily or even exclusively in trials preceded by a congruent trial, we also calculated the retest reliability separately for these two conditions. In fact, for trials preceded by a previous congruent stimulus, the correlation was moderately positive but still not quite significant, *r* = 0.25, (*r*_c_ = 0.40), *t*(46) = 1.73, *p* = 0.10; but for trials preceded by a previous incongruent stimulus, the correlation was numerically even negative, *r* = − 0.23, *t*(46) = 1.59, *p* = 0.13. The correlation of the sequential congruency effect (computed as congruency effect after congruent minus congruency effect after incongruent trials) was *r* = − 0.23, *t*(46) = 1.64, *p* = 0.12. Hence, in RT data, neither the congruency effect, nor the sequential congruency effect, significantly correlated between Session 1 and Session 2, thus showing no significant retest reliability even if sessions were separated by only 10 min. of an intervening filler task.

In error data, the retest reliability of the congruency effect (averaged across previous congruency) was *r* = 0.45, *t*(46) = 3.40, *p* < 0.01. For trials preceded by a previous congruent stimulus, the correlation was *r* = 0.55, (*r*_c_ = 0.71), *t*(46) = 4.50, *p* < 0.01; and again, for trials preceded by a previous incongruent stimulus, the correlation was nearly eliminated, *r* = 0.10, (*r*_c_ = 0.19), *t*(46) < 1. The retest reliability of the sequential congruency effect was *r* = 0.18, *t*(46) = 1.25, *p* = 0.22. That is, in error data, there was a significant retest reliability of the congruency effect, but primarily for the congruency effect after congruent trials. The congruency effect after incongruent trials, as well as the sequential congruency effect, were reliable, consistent with the lack of retest reliability in RT.

#### Comparison of correlation coefficients

The important new finding that split-half and retest reliability of the congruency effect was larger for trials *after* congruent than for trials *after* incongruent trials was further investigated statistically, using the “cocor” web interface (http://comparingcorrelations.org; Diedenhofen & Musch, 2015). Because the different statistics all yielded very similar results, we only report the most conservative statistic of Silver et al.’s modification of Dunn and Clark’s *z* value, as well as Zou’s 95% confidence interval. All tests were two-tailed.

Retest reliability of the RT congruency effect was significantly larger for trials *after* congruent trials than for trials *after* incongruent trials (Silver et al.’s *z* = 2.3071, *p* = 0.02; Zou’s 95% confidence interval: [0.08, 0.83]). Split-half reliability of the RT congruency effect was also significantly larger for trials *after* congruent trials than for trials *after* incongruent trials (Silver et al.’s *z* = 2.7911, *p* = 0.005; Zou’s 95% confidence interval: [0.16, 0.88]).

For the congruency effect in error rates, retest reliability was significantly larger for trials *after* congruent than for trials *after* incongruent trials (Silver et al.’s *z* = 2.5392, *p* = 0.01; Zou’s 95% confidence interval: [0.10, 0.78]), whereas split-half reliability did not statistically differ between the two conditions (Silver et al.’s *z* = 0.6471, n.s.; Zou’s 95% confidence interval: [− 0.22, 0.44]). The descriptive statistics of the distributions of congruency effects and conflict-adaptation effects are summarized in Table 1.

#### Bootstrapping analysis of split-half reliability

For the combined data of both sessions, the median bootstrapped reliability estimates were similar to the respective odd–even split-half reliability estimates: For the congruency effect, reliabilities were around *r* ~ 0.30 in RT (*r*_median_ = 0.27; 95% range from *r* = − 0.04 to *r* = 0.51; versus *r*_odd–even_ = 0.34), and around *r* ~ 0.50 in error rates (*r*_median_ = 0.51; 95% range from *r* = 0.32 to *r* = 0.66; versus *r*_odd–even_ = 0.53). For the sequential congruency effect, split-half reliability was virtually zero in both RT (*r*_median_ = − 0.17; 95% range from *r* = − 0.47 to *r* = 0.15; versus *r*_odd–even_ =  − 0.01 for the odd–even reliability) and error rates (*r*_median_ = 0.12; 95% range from *r* =  − 0.11 to *r* = 0.34; versus *r*_odd–even_ = 0.20).

### Discussion

In Experiment 2, we found the typical congruency effect and the sequential congruency effect that is known from the literature (see Egner, 2017; Duthoo et al., 2014) and we replicated our own previous findings using the same experimental Stroop-like paradigm (e.g., Schuch & Koch, 2015). The novel aspect of the present experiment was the calculation of split-half and retest reliability of these experimental effects.

Interestingly, reliability scores for the congruency effect and the sequential congruency effect were somewhat larger in error data than in RT data. This is different from the reliability scores of CSI effect and *N *− 2 repetition costs in Experiment 1, which were larger in RT than in error data, and suggests that for (sequential) congruency effects, error rates should be considered in addition to RT data.

Regarding the size of the reliability scores, previous studies have reported quite high reliability of the Stroop effect (e.g., Friedman & Miyake, 2004, using a color-word Stroop effect with vocal responses). In the present study, the split-half reliability of the Stroop-like effect was moderate at best (*r* = 0.34 and *r*_median_ = 0.27 in RT data, *r* = 0.53 and *r*_median_ = 0.51 in error data for odd–even and bootstrapped reliabilities, respectively). The finding of lower reliability, along with an overall smaller size of the Stroop-like effect, might be due to several reasons. First, we used manual instead of vocal responses, and the task required a categorization of the stimulus (i.e., categorizing the name as female or male) instead of an identification of stimulus (i.e., identifying the ink color). Second, we applied strict filtering of the data, excluding all trials that constituted partial feature repetitions with the preceding trial, to eliminate any trial-to-trial effects of episodic interference. This filtering procedure is especially important for the sequential congruency effect (to obtain a “pure” measure of conflict adaptation), but might also attenuate the size of the congruency effect.

Notably, when we restricted our reliability analyses to trials preceded by congruent trials (i.e., to those trials in which the congruency effect is larger), then we found a higher correlation (odd–even split-half reliability was *r*_c_ = 0.69 in RT data, *r*_c_ = 0.62 in error data), indicating that these trials are probably better suited to assess the split-half reliability of the Stroop effect (in RT data, the uncorrected split-half reliability scores were significantly larger for trials preceded by congruent vs incongruent trials; in error data, this difference did not reach statistical significance).

Retest reliability was low for the RT effect (*r* = 0.16), but was similar to split-half reliability for the error data (*r* = 0.45 in error data). When only trials after congruent trials were considered, retest reliability of the Stroop-like effect in error data was good (*r*_c_ = 0.71), suggesting that Stroop-like interference effects should be assessed in error data in addition to RT data, and should be restricted to trials after congruent trials when used for interindividual-difference approaches.

Compared to the Stroop-like congruency effect itself, the sequential modulation of the congruency effect did not show a significant retest reliability, confirming recent demonstrations of lacking reliability of sequential modulations of the “family of conflict effects”, such as the Stroop effect, the Simon effect, and the Flanker effect (see Whitehead et al., 2019, for a recent discussion). Note that we carefully controlled for episodic retrieval effects, excluding all trials with partial feature repetitions from analysis. This rather strict way of data filtering was applied to get a measure of conflict adaptation that is not (or only minimally) “contaminated” by episodic interference effects. This rather strict way of filtering leads to smaller congruency effects and sequential congruency effects (cf. Whitehead et al., 2019), which in turn might have contributed to the rather low reliability scores in the present experiment.

## General discussion

In this study, we examined the reliability of four common measures of cognitive control. In Experiment 1, we reported two task-switching measures: N-2 task repetition costs as a marker of task-level inhibition, and the influence of task-preparation time (i.e., CSI variation) as a marker of cue-based task preparation. In Experiment 2, we assessed two cognitive-control measures from single-task paradigms: Applying a Stroop-like face–name interference paradigm, we examined the congruency effect, which is a measure of cognitive conflict, as well as the sequential congruency effect, which can be related to conflict-triggered control adjustments. In both experiments, we observed the expected experimental effects on the group level. In Experiment 1, we found quite sizeable *N *− 2 task repetition costs and a substantial cue-based task preparation effect. Moreover, *N *− 2 task repetition costs were reduced with long preparation time. In Experiment 2, we found a clear congruency effect and a significant sequential congruency effect, while controlling for episodic influences on these effects.

The novel findings pertain to the reliability analyses. For the CSI effect, split-half and retest reliability was good. For *N *− 2 repetition costs, split-half and retest reliability was poor. For the Stroop-like congruency effect, split-half and retest reliabilities were moderate, but were improved when focusing on trials after congruent trials (where a larger congruency effect occurs). For the conflict adaptation effect, reliabilities were poor. In the following, we discuss these findings in some more detail, and then turn to methodological considerations.

### Reliability of conflict adaptation effect and congruency effect

The finding of lacking reliability of sequential modulations of Stroop-like congruency effects has recently been thoroughly investigated by Whitehead et al. (2019). Our data confirm their findings and further extend them to lacking retest reliability (which does not come as a surprise given that split-half reliability was not significant in the first place).

In comparison to the sequential congruency effect, the congruency effect itself (i.e., the face-name Stroop effect) had a reasonable reliability, especially when focusing on error rates. This reliability can be even improved if the calculation of reliability is restricted to those conditions in which a strong congruency effect is expected, that is, *after* congruent trials. Hence, one practical implication of the present study is that a more reliable measure of conflict processing can be derived when the data are partitioned into trials following congruent vs. incongruent trials.

### Reliability of task-preparation effect and task-inhibition effect

The cue-based task preparation effect showed the highest reliability scores of the four cognitive-control measures investigated here, suggesting it is a good candidate for future investigations of cognitive control using correlational approaches. As the present task-switching paradigm consisted of task switches only, we suggest that the task-preparation effect as measured here may be taken as a measure of participants’ efficiency of cue-based activation of task set.

Another important result of the present study is the lacking retest reliability of *N *− 2 task repetition costs in Experiment 1, while at the same time the *N *− 2 repetition costs were highly robust as an experimental effect in both sessions. This might be a case of the “reliability paradox” as discussed by Hedge et al., (2018b), who argued that very strong effects at the level of group means are often counterintuitively less reliable at the level of interindividual differences, because if all participants display an effect of similar size (i.e. in very homogeneous groups), already small intraindividual variations can substantially attenuate the correlation across participants (see also Paap & Sawi, 2016, for a discussion).

An interesting question pertains to the interpretation of this lacking retest reliability of the task-inhibition effect. Does it mean that a trait-like cognitive ability of task inhibition does not exist? Alternatively, does it mean that we cannot measure trait-like inhibitory ability on an interindividual-differences level, because our *N *− 2 repetition cost measure is too noisy?

One possibility is that inhibitory control is not stable within an individual, but is highly state-dependent, and is applied in a context-sensitive way (cf. Rey-Mermet et al., 2018). If so, it would be quite plausible to assume a substantial fluctuation of inhibitory control across trials. The case could be similar as with sequential fluctuation of Stroop-like effects: Stroop-like effects are usually more pronounced after no-conflict (i.e., congruent) than after conflict (i.e., incongruent) trials, which might reflect trial-to-trial fluctuations of attentional selectivity. Specifically, after no-conflict trials, the cognitive system is in a state of unfocused attention, so that the irrelevant stimulus dimension is processed up to the level of response activation, which in the case of incongruent stimuli results in a response conflict and triggers an upregulation of selective attention. In contrast, after conflict trials, the system is in a state of highly selective attention, so that no irrelevant response is activated, hence there is no response conflict, and no further upregulation of selective attention occurs. That is, for Stroop-like effects, the demand for an upregulation of cognitive control differs greatly across trials depending on the conflict level of the preceding trial. A similar scenario is conceivable for task-level inhibition: The demand for inhibitory task-level control might depend on the degree of task conflict in a particular trial. There is evidence that the amount of task-level inhibition (as measured by the size of *N *− 2 repetition costs) depends on the degree of task-set competition. Conditions with low task-set competition, such as when previous task-set activation has decayed (Gade & Koch, 2005) or when the current task set has been very well prepared (e.g., Scheil & Kleinsorge, 2014), have been shown to attenuate *N *− 2 task repetition costs. That is, fluctuations of activation level of the current task and of competing tasks jointly determine the degree of inhibitory control. In that sense, inhibition is not a process that is mandatory in every trial and always to the same degree but is a highly context-dependent, adaptive process. Hence, if this “inhibition on demand” account were correct, we would expect low intraindividual stability, which is reflected in low reliability, even though, when aggregated across many trials, there is clear evidence for such an effect at the individual and group level. This “inhibition on demand” idea could explain the discrepancy between reliability measures and group-level effects; however, it cannot account for the observed discrepancy between moderate split-half reliability and near-zero retest reliability of *N *− 2 repetition costs observed in the present study.

### Methodological considerations

Of the four cognitive-control measures investigated here, the largest effect (i.e., the task-preparation effect) showed the best reliability scores. This might be due to methodological reasons. First, the higher reliability of the task-preparation effect as compared to that of the other effects might be partly due to its larger *effect size*. For instance, in the present Experiment 1, the overall mean CSI effect was 284 ms, with a standard deviation [SD] of 98 ms, corresponding to Cohen’s d effect size of 2.89; *N *− 2 repetition costs, on the other hand, were 43 ms, with SD of 37 ms, corresponding to Cohen’s *d* of 1.14. When only looking at Session 1, the variability of the CSI effect across participants (SD = 112 ms in Session 1) relative to its size (mean CSI effect = 305 ms in Session 1) is smaller than the variability of *N *− 2 repetition costs across participants (SD = 47 ms in Session 1) relative to their size (mean *N *− 2 repetition costs = 50 ms in Session 1). Therefore, the correlation of the CSI effect between Sessions 1 and 2 can be higher than the correlation of *N *− 2 repetition costs between Sessions 1 and 2.

Second, apart from effect size, the *absolute size* of an effect may also play a role for reliability. For instance, the CSI effect observed in the present study amounted to about 280 ms, whereas *N *− 2 repetition costs were in the order of magnitude of 40 ms. The larger absolute size of an effect could contribute to its reliability scores. This is because the unsystematic measurement error inherent to computer hardware and software constitutes a lower limit to the reliability of cognitive effects that are small in absolute size (e.g., 50 ms or smaller). The unsystematic error of a single-trial RT measurement depends on hardware and software settings, and may be in the order of 20 ms or larger (e.g., Plant & Turner, 2009; Plant et al., 2004). This means that also the average scores per condition, and the difference scores between conditions, are subject to unsystematic measurement error (the more trials are included for averaging, the smaller the unsystematic error). The measurement error is also reflected in the variability of the difference scores across participants. This constitutes a lower limit to the reliability with which smaller effects can be measured (such as *N *− 2 repetition costs, which were about 40 ms in the present experiment). For larger effects (such as the CSI effect, which was about 300 ms in the present experiment, or task-switch costs, which are often around 200 ms to 300 ms), the variability due to technical timing inaccuracy is proportionally smaller (given the same number of trials included for averaging), such that effects that are larger in absolute size (e.g., 200 ms or larger) can be measured with higher reliability. Consistent with this reasoning, for instance, Whitehead et al. (2019) observed good reliability of the post-error slowing effect (an effect of around 100 ms, 160 ms, and 300 ms in their Experiments 1, 2, and 3, respectively), but poor reliability of the sequential congruency effect (which was about 10 ms in their Experiments 1 and 2, and about 14 ms in their Experiment 3). Likewise, Friedman and Miyake (2004) observed good split-half reliability of large effects (e.g., task-switch costs that were in the range of 200–500 ms), moderate reliability of smaller effects (e.g., residual switch costs of their category switching paradigm that were about 70 ms), and poor reliability of very small effects (e.g., negative priming effects that were in the range of 3–8 ms). Future research would need to investigate systematically the relationship between the absolute size of an effect and the maximum reliability that can be achieved for this effect.

Another issue that complicates the measurement of trait-like cognitive control abilities are recent observations that difference scores in cognitive-control tasks are not “pure” measures of control (e.g., of conflict processing), but also reflect settings of individual speed-accuracy tradeoff (SAT) and general processing speed (Hedge et al., 2018a, 2021).﻿

Using computer simulations and computational modeling, Hedge and colleagues (2018a, 2021) demonstrated that correlations between different cognitive-control measures (e.g., between Stroop effect and Flanker effect) can be observed even if there are no correlations between the model parameters reflecting conflict processing, but only correlations between model parameters reflecting SAT setting and/or general processing speed. Moreover, if there *are* correlations between the conflict-related model parameters across the different tasks, this does not necessarily lead to correlations between the behavioral difference scores in the two tasks. These findings call into question the widespread assumption in the literature that computing difference scores would “cancel out” interindividual differences in SAT settings and processing speed.

### Conclusion

Together, the data suggest that all four cognitive control measures investigated here (task-inhibition effect, task-preparation effect, Stroop-like effect, and conflict adaptation effect) are well-suited for assessing group-level effects of cognitive control. Yet, except for the task-preparation effect, these measures do not seem suitable for reliably assessing interindividual differences in the strength of cognitive control. Therefore, they do not seem suited for correlational approaches, such as structural equation modeling, assessment of correlations with psychophysiological data (EEG, fMRI, etc.), or correlations with psychometric constructs (e.g., questionnaire data assessing rumination tendency). In line with recent claims in the field (Parsons et al., 2019), we suggest that researchers should assess and optimize reliability of their behavioral measures before subjecting them to correlational analyses.

## Supplementary Information

Below is the link to the electronic supplementary material.

Supplementary file1 (DOCX 95 kb)

## Data Availability

The raw data of this article are available at the following link: https://osf.io/dga2c/?view_only=2e4cb78f22114dcaabc1d9b92c1ee5de.

## Footnotes

1 The General Spearman-Brown correction for reducing trial numbers by a factor of N is r_c_ = N*r/[1 + [N-1]*r]. For halving of trial numbers (i.e. reducing trial numbers by a factor of 2) it is therefore r_c_ = 2*r/[1 + r].

2 Due to experimenter error, two participants who were supposed to start with short CSI started with long CSI instead (in Session 1 only). Moreover, one of these participants received different response mappings in Session 1 and Session 2. The overall data pattern was very similar when these participants’ data were included or excluded, so we included them in the sample.

3 Two different filler tasks were used, manipulated between participants, and orthogonally to response mapping and CSI order. For the main analysis reported here, we collapsed data analysis across both filler-task groups. For analyses with filler-task group as additional factor, see Supplementary Material. We also used two standardized questionnaires (see Supplementary Material for further description and summary of correlations between behavioral and questionnaire measures).

4 To check for the robustness of the estimates from the bootstrapping procedure with 1,000 iterations, we performed each bootstrapping procedure twice. The estimates were almost identical for the first and second run, suggesting that 1,000 iterations were enough to produce robust estimates. Therefore, we only report the estimates of the first run.

5 Due to onset of the COVID-19 pandemia, we were not able to collect data from 96 participants.

6 As with Experiment 1, we used two different filler tasks, manipulated between participants, and orthogonally to response mapping. In the analyses reported here, the data were collapsed across both groups; see Supplementary Material for analyses with filler-task group as additional factor. We also used the same two questionnaires as in Experiment 1; see Supplementary Material for a summary of correlations between behavioral and questionnaire measures.
