# Supplementary material for: On the reliability of behavioral measures of cognitive control: retest reliability of task-inhibition effect, task-preparation effect, Stroop-like interference, and conflict adaptation effect
Source: Psychol Res. 2021 Dec 18;86(7):2158–84. doi: 10.1007/s00426-021-01627-x (PMC8683338; doi:10.1007/s00426-021-01627-x)
Supplement: Supplementary file 1 — Supplementary file1 (DOCX 95 kb) [file 426_2021_1627_MOESM1_ESM.docx]

**SUPPLEMENTARY MATERIAL**

On the reliability of behavioral measures of cognitive control: Retest reliability of task-inhibition effect, task-preparation effect, Stroop-like interference, and conflict adaptation effect

Authors: Stefanie Schuch, Andrea M. Philipp, Luisa Maulitz & Iring Koch

**Overview of Supplementary Material:**

**I. Description of the additional between-subjects factor and the questionnaire measures**

**II. Analyses including the factor group (filler task: mindfulness task vs. crossword puzzle task)**

**III. Correlations of questionnaire scores (DESC and FMI) with the behavioral effects**

**IV. References**

**I. Description of the additional between-subjects factor and the questionnaire measures**

The original purpose of the study was to assess the influence of a short mindfulness intervention on cognitive control processes. To this end, half of the participants received a 10-minutes guided mindfulness meditation for focusing attention (see Hoelzel et al., 2011), whereas the other half solved a crossword puzzle during the 10-minute break. The factor group was manipulated orthogonally to response mapping and CSI order.

Related to the original research question, participants also filled in the German versions of two questionnaires: The DESC (see Forkmann et al. 2010), measuring depression tendency, prior to the experiment, and the FMI (Walach et al., 2006), measuring the degree of mindfulness, after the second session. We did not observe any significant correlations between questionnaire data and behavioral measures (see below). Neither did we observe any differences between the groups, except for one effect in error rates, which however was not mirrored in RT data: in error rates, the effect of CSI was larger in the mindfulness group than control group (see below for an ANOVA comparing performance between groups, and for Figures plotting the data separately for the two groups). For the analysis reported in the main paper, the data were collapsed across both groups.

**II. Analyses including the factor group (filler task: mindfulness task vs. crossword puzzle task)**

Table S1. Experiment 1: Analysis of Variance (ANOVA) on RT data.

|  | F(1,94) | p value | partial eta square |
| --- | --- | --- | --- |
| Group | 0.03 | 0.58 | 0.00 |
| Session | 61.5 | 0.00 | 0.40 |
| Session * Group | 3.26 | 0.07 | 0.03 |
| CSI | 809.5 | 0.00 | 0.90 |
| CSI * Group | 1.98 | 0.16 | 0.02 |
| Task sequence | 124.3 | 0.00 | 0.57 |
| Task sequence * Group | 0.03 | 0.87 | 0.00 |
| CSI * Task sequence | 11.30 | 0.00 | 0.11 |
| CSI * Task sequence * Group | 0.77 | 0.38 | 0.01 |
| Session * CSI | 15.36 | 0.00 | 0.14 |
| Session * CSI * Group | 0.90 | 0.35 | 0.01 |
| Session * Task sequence | 5.29 | 0.02 | 0.05 |
| Session * Task sequence * Group | 0.09 | 0.76 | 0.00 |
| Session * CSI * Task sequence | 1.19 | 0.28 | 0.01 |
| Session * CSI * Task sequence * Group | 0.01 | 0.91 | 0.00 |

Table S2. Experiment 1: Analysis of Variance (ANOVA) on Error data.

|  | F(1,94) | p value | partial eta square |
| --- | --- | --- | --- |
| Group | 1.32 | 0.25 | 0.01 |
| Session | 13.43 | 0.00 | 0.13 |
| Session * Group | 2.39 | 0.13 | 0.03 |
| CSI | 3.84 | 0.05 | 0.04 |
| CSI * Group | 4.86 | 0.03 | 0.05 |
| Task sequence | 17.26 | 0.00 | 0.16 |
| Task sequence * Group | 0.01 | 0.93 | 0.00 |
| CSI * Task sequence | 0.01 | 0.94 | 0.00 |
| CSI * Task sequence * Group | 0.28 | 0.60 | 0.00 |
| Session * CSI | 2.11 | 0.15 | 0.02 |
| Session * CSI * Group | 2.29 | 0.13 | 0.02 |
| Session * Task sequence | 0.36 | 0.55 | 0.00 |
| Session * Task sequence * Group | 2.19 | 0.14 | 0.02 |
| Session * CSI * Task sequence | 1.18 | 0.28 | 0.01 |
| Session * CSI * Task sequence * Group | 0.04 | 0.85 | 0.00 |

Figure S1. Experiment 1. Mean RT as a function of Session (Session 1, Session 2), Task Sequence (ABA, CBA), and CSI (100 ms, 900 ms), separately for the groups with different filler tasks between Sessions 1 and 2 (mindfulness intervention vs. crossword puzzle). Error bars indicate one standard error of mean.

**Group with mindfulness intervention between sessions**

**Group with crossword puzzle between sessions**

Figure S2. Experiment 1. Mean Error Rate as a function of Session (Session 1, Session 2), Task Sequence (ABA, CBA), and CSI (100 ms, 900 ms), separately for the groups with different filler tasks between Sessions 1 and 2 (mindfulness intervention vs. crossword puzzle). Error bars indicate one standard error of mean.

**Group with mindfulness intervention between sessions**

**Group with crossword puzzle between sessions**

Table S3. Experiment 2: Analysis of Variance (ANOVA) on RT data.

|  | F(1,46) | p value | partial eta square |
| --- | --- | --- | --- |
| Group | 0.29 | 0.60 | 0.01 |
| Session | 28.89 | 0.00 | 0.39 |
| Session * Group | 2.22 | 0.14 | 0.05 |
| PreviousCongruency | 13.90 | 0.00 | 0.23 |
| PreviousCongruency * Group | 1.53 | 0.22 | 0.03 |
| Congruency | 241.06 | 0.00 | 0.84 |
| Congruency * Group | 0.11 | 0.75 | 0.00 |
| PreviousCongruency * Congruency | 6.64 | 0.01 | 0.13 |
| PreviousCongruency * Congruency * Group | 0.00 | 0.95 | 0.00 |
| Session * PreviousCongruency | 0.04 | 0.84 | 0.00 |
| Session * PreviousCongruency * Group | 0.78 | 0.38 | 0.02 |
| Session * Congruency | 5.99 | 0.02 | 0.12 |
| Session * Congruency * Group | 0.06 | 0.80 | 0.00 |
| Session * PreviousCongruency * Congruency | 0.01 | 0.92 | 0.00 |
| Session * PreviousCongruency * Congruency * Group | 0.27 | 0.60 | 0.01 |

Table S4. Experiment 2: Analysis of Variance (ANOVA) on Error data.

|  | F(1,46) | p value | partial eta square |
| --- | --- | --- | --- |
| Group | 0.18 | 0.67 | 0.00 |
| Session | 2.34 | 0.13 | 0.05 |
| Session * Group | 1.95 | 0.17 | 0.04 |
| PreviousCongruency | 3.46 | 0.07 | 0.07 |
| PreviousCongruency * Group | 5.80 | 0.02 | 0.11 |
| Congruency | 69.91 | 0.00 | 0.60 |
| Congruency * Group | 1.21 | 0.28 | 0.03 |
| PreviousCongruency * Congruency | 8.09 | 0.01 | 0.15 |
| PreviousCongruency * Congruency * Group | 0.04 | 0.84 | 0.00 |
| Session * PreviousCongruency | 0.00 | 1.00 | 0.00 |
| Session * PreviousCongruency * Group | 0.77 | 0.39 | 0.02 |
| Session * Congruency | 0.05 | 0.83 | 0.00 |
| Session * Congruency * Group | 0.40 | 0.53 | 0.01 |
| Session * PreviousCongruency * Congruency | 2.88 | 0.10 | 0.06 |
| Session * PreviousCongruency * Congruency * Group | 0.05 | 0.83 | 0.00 |

**III. Correlations of questionnaire scores (DESC and FMI) with the behavioral effects**

Table S5. Experiment 1. Correlations of questionnaire scores (DESC and FMI) with behavioral effects (N-2 repetition costs and CSI effect; N-2 repetition costs were averaged across sessions and CSI conditions; the CSI effect was averaged across sessions and task sequences). N=96.

|  | N-2 repetition costs in RT | N-2 repetition costs in Error Rates | CSI effect in RT | CSI effect in Error Rates |
| --- | --- | --- | --- | --- |
| DESC |  |  |  |  |
| correlation r | 0.10 | -0.03 | 0.04 | 0.04 |
| t(94) | 0.94 | 0.31 | 0.38 | 0.35 |
| p two-tailed | 0.35 | 0.76 | 0.70 | 0.73 |
| FMI |  |  |  |  |
| correlation r | 0.08 | 0.03 | -0.08 | 0.01 |
| t(94) | 0.79 | 0.27 | 0.74 | 0.09 |
| p two-tailed | 0.43 | 0.79 | 0.46 | 0.93 |

Table S6. Experiment 2. Correlations of questionnaire scores (DESC and FMI) with Sbehavioral effects (congruency effect and sequential congruency effect; the congruency effect was averaged across sessions and previous-trial congruency levels; the sequential congruency effect was averaged across sessions). N=48.

|  | Congruency effect in RT | Congruency effect in Error Rates | Sequential congruency effect in RT | Sequential congruency effect in Error Rates |
| --- | --- | --- | --- | --- |
| DESC |  |  |  |  |
| correlation r | 0.26* | -0.27* | 0.04 | -0.17 |
| t(46) | 1.82 | 1.93 | 0.26 | 1.19 |
| p two-tailed | 0.07 | 0.06 | 0.79 | 0.24 |
| FMI |  |  |  |  |
| correlation r | -0.18 | 0.07 | -0.02 | 0.17 |
| t(46) | 1.27 | 0.47 | 0.11 | 1.15 |
| p two-tailed | 0.21 | 0.64 | 0.91 | 0.26 |

Note. The two correlations indicated by an asterisk were driven by the data points from the three participants with the largest DESC values; when these three data points were excluded, the correlations dropped to r=.12 and r=-.03 for the congruency effect in RT and error data, respectively.

**IV. References**

Hoelzel, B. K., Lazar, S. W., Gard, T., Schuman-Olivier, Z., Vago, D. R., & Ott, U. (2011). How does mindfulness meditation work? Proposing mechanisms of action from a conceptual and neural perspective. *Perspectives on Psychological Science, 6*, 537-559. doi: 10.1177/1745691611419671

Forkmann, T., Boecker, M., Wirtz, M., Glaesmer, H., Brahler, E., Norra, C., & Gauggel, S. (2010). Validation of the Rasch-based Depression Screening in a large scale German general population sample. *Health and Quality of Life Outcomes, 8*, 105. doi: 10.1186/1477-7525-8-105

Walach, H., Buchheld, N., Buttenmüller, V., Kleinknecht, N., & Schmidt, S. (2006). Measuring mindfulness – The Freiburg Mindfulness Inventory (FMI). *Personality and Individual Differences, 40*, 1543-1555. doi: 10.1016/j.paid.2005.11.025
